# Prophylactic treatment with broadly neutralizing antibody VRC01 selects for escape mutants after infection in Antibody-Mediated Prevention trials

**DOI:** 10.1038/s41564-026-02467-4

**Published:** 2026-09-28

**Authors:** Carolyn Williamson, Lyle Curry, Nonhlanhla N. Mkhize, Elena E. Giorgi, Craig A. Magaret, Bronwen E. Lambson, Sinethemba Bhebhe, Haajira Kaldine, Thandeka Moyo-Gwete, Morgane Rolland, Raabya Rossenkhan, Nina Marie G. Garcia, Chivonne Moodley, Anna Yssel, Yunda Huang, Alaine A. Marsden, Daniel B. Reeves, Bryan T. Mayer, Roger E. Bumgarner, Nicolas Beaume, Dylan H. Westfall, Michal Juraska, Allan C. DeCamp, Hugh Murrell, Hongjun Bai, Wenjie Deng, Alec P. Pankow, Tanmoy Bhattacharya, Talita York, Nonkululeko Ndabambi, Lennie Chen, Hong Zhao, Asanda Gwashu-Nyangiri, Ruwayhida Thebus, Paula Cohen, Ben Murrell, Shelly Karuna, John Hural, Nyaradzo Mgodi, Srilatha Edupuganti, Lynn Morris, David Montefiori, M. Juliana McElrath, Myron S. Cohen, Lawrence Corey, Paul T. Edlefsen, Peter B. Gilbert, Penny L. Moore, James I. Mullins

**Affiliations:** 1 Division of Medical Virology, Institute of Infectious Disease and Molecular Medicine and Wellcome CIDRI-Africa Centre, Faculty of Health Sciences, University of Cape Town, Cape Town, South Africa; 2 National Health Laboratory Service, Johannesburg, South Africa; 3 Centre for the AIDS Programme of Research in South Africa, University of Kwa-Zulu Natal, Durban, South Africa; 4 SA MRC Antibody Immunity Research Unit, School of Pathology, Faculty of Health Sciences, University of the Witwatersrand, Johannesburg, South Africa; 5 National Institute for Communicable Diseases of the National Health Laboratory Service, Johannesburg, South Africa; 6 Vaccine and Infectious Disease Division, Fred Hutchinson Cancer Center, Seattle, WA USA; 7 US Military HIV Research Program, CIDR, Walter Reed Army Institute of Research, Silver Spring, MD USA; 8 Henry M. Jackson Foundation for the Advancement of Military Medicine, Bethesda, MD USA; 9 Department of Global Health, University of Washington, Seattle, WA USA; 10 Department of Microbiology, University of Washington, Seattle, WA USA; 11 Los Alamos National Laboratory, Los Alamos, NM USA; 12 Santa Fe Institute, Santa Fe, NM USA; 13 Department of Microbiology, Tumor and Cell Biology, Karolinska Institute, Solna, Sweden; 14 University of Zimbabwe College of Health Sciences Clinical Trials Research Centre, Harare, Zimbabwe; 15 Department of Medicine, Division of Infectious Diseases, Emory University School of Medicine, Atlanta, GA USA; 16 Department of Surgery, Duke University Medical Center, Durham, NC USA; 17 Department of Medicine, University of North Carolina at Chapel Hill, Chapel Hill, NC USA; 18 Wits Infectious Diseases and Oncology Research Institute, Faculty of Health Sciences, University of the Witwatersrand, Johannesburg, South Africa; 19 Present Address: Vaccine and Infectious Disease Division, Fred Hutchinson Cancer Center, Seattle, WA USA

**Keywords:** Viral evolution, HIV infections

## Abstract

Broadly neutralizing antibodies (bnAbs) show promise in HIV prevention, yet viral escape remains a challenge. In the Antibody-Mediated Prevention (AMP) trials, the CD4 binding site (CD4bs) bnAb VRC01 blocked acquisition by VRC01-sensitive strains. However, its influence on viral evolution post acquisition is not fully understood. Here we analysed 11,730 HIV envelope sequences generated during the AMP trials from 47 participants, identifying VRC01-mediated de novo escape mutations in 8 of 26 VRC01-treated participants but none in 21 placebo participants. These mutations were found at very low frequencies (<1%) in globally circulating viruses, suggesting that they may confer fitness costs. Escape mutations, primarily located in the Loop-D and β23/V5 regions of Env, conferred cross-resistance to several CD4bs bnAbs; however, more potent CD4bs bnAbs such as N6 and 1–18 largely retained their activity. Our findings demonstrate that prophylactic VRC01 can select for viral escape after infection, underscoring the need for next-generation bnAbs with improved breadth and potency to enhance durability and efficacy of antibody-based HIV prevention.

## Abstract (web summary)

Prophylactic bnAb VRC01 selected for escape mutations in 31% of HIV breakthrough infections, but these mutations were rare in circulating viruses suggesting a fitness cost. More potent bnAbs such as N6 and 1-18 largely retained activity against escape variants.

## Main

Broadly neutralizing antibodies (bnAbs) play a key role in both vaccine and passive immunization prevention strategies^1,2^. The Antibody-Mediated Prevention (AMP) trials were two harmonized phase 2b efficacy trials that tested whether the CD4 binding site (CD4bs) antibody VRC01 could prevent HIV acquisition^3^. The study recruited 4,632 at-risk women in sub-Saharan Africa (HVTN 703/HPTN 081) and at-risk cisgender men and transgender persons in North and South America and in Europe (HVTN 704/HPTN 085). These landmark studies demonstrated that passively administered bnAbs could prevent HIV acquisition: VRC01 was 75% effective against highly sensitive viruses (IC_80_ < 1 µg ml^−1^)^3^, where IC_80_ is the antibody concentration needed to inhibit infection by 80%, and serum neutralization titres were a reliable biomarker of protection^4^. However, efficacy declined with increased viral resistance and greater genetic divergence in the VRC01 epitope^3,5^.

VRC01 transiently suppressed viral load in individuals who acquired susceptible viruses^3,6^, and in those with multilineage infections involving both sensitive and resistant strains, the sensitive viruses declined over time^7^. This post-acquisition bnAb activity raises concerns that bnAb-based prevention strategies may select for viral resistance which could reduce the effectiveness of future antibody-mediated interventions and vaccines.

Mapping VRC01 resistance is challenging because the epitope is quaternary (located across multiple subunits of the viral envelope). Resistance-associated mutations have been identified across gp120, including in Loop D, the CD4 binding loop, β20/β21, β23 and the V5 loop^8–13^, but can also arise in distal sites outside these structurally defined VRC01 contact points^9,14^. Although bnAb resistance mutations confer a selective advantage to the virus under antibody pressure, they are often found at very low frequency among circulating viruses and can incur a fitness cost^15,16^.

Viral escape is determined by the interplay of viral features, including sequence diversity and the fitness cost of escape mutations, and host factors such as the specificity and magnitude of the immune response^17–19^. During bnAb-mediated prevention, antibodies are present at the time of acquisition when viral diversity is typically very low^20,21^. Consistent with a high barrier to escape under these conditions, no viral evolution was observed in rebound virus from individuals who received anti-retroviral therapy (ART) during acute infection and later switched to VRC01 immunotherapy^22^. In contrast, individuals treated with ART during chronic infection experienced rapid viral rebound following VRC01 immunotherapy, driven by either pre-existing resistant mutations or high diversity of the reservoirs, which provides shorter evolutionary pathways to escape^23–25^. The AMP trials therefore provided a unique opportunity to investigate viral escape under bnAb pressure immediately following acquisition, before substantial viral diversification, a dynamic that is difficult to disentangle in therapeutic settings, where resistance can arise from archived reservoir viruses^26–28^.

Although resistance has been documented in ART-mediated pre-exposure prophylaxis (PrEP)^29^, the AMP trial was the first opportunity to evaluate whether bnAb-mediated PrEP selects for viral escape following HIV acquisition, and if so, whether such mutations compromise the effectiveness of other clinically relevant CD4 binding site bnAbs. To study post-acquisition escape in participants who acquired VRC01-susceptible viruses, we analysed 11,730 HIV envelope (*Env*) sequences and tracked evolution in the VRC01 epitopes from a single founder lineage. This approach allowed us to detect escape present at very low frequencies. Although several mutations conferred cross-resistance to certain CD4bs bnAbs, the more potent bnAbs generally remained effective.

## Results

### Participants with VRC01 single-lineage infections (IC_80_ ≤ 3 µg ml^−1^)

AMP study participants received infusions of saline (placebo), 10 mg kg^−1^ (low-dose) or 30 mg kg^−1^ (high-dose) VRC01 every 8 weeks, with monthly HIV diagnostic testing^3^. Infections were classified into single or multiple transmitted founder lineage (TFL) based on deep sequencing of the *env* and *gag* gene from the earliest RNA-positive plasma sample. Pseudoviruses (PSVs) representing the TFL, and neutralization data linked to these lineages, were available^3^.

To identify post-acquisition VRC01-driven viral evolution from a single TFL sequence, we examined viral sequences from 47 participants who acquired a single TFL with low to intermediate VRC01 resistance (IC_80_ ≤ 3 µg ml^−1^)^3,7^. Twenty-three were from the Africa trial and 24 from the Americas trial, with 21 participants from the placebo group, 15 from the low-dose group and 11 from the high-dose group (Table 1). Thirty-nine were diagnosed by the week 80 visit (within ±8 weeks of the final infusion, trial endpoint) and 8 were diagnosed after the week 80 visit. We only included sequences from participants sampled within 5 weeks of estimated date of acquisition^30^ to minimize sequence changes driven by host CD4bs responses^30,31^. Inclusion of placebo participants controlled for changes in VRC01 sensitivity attributed to host immune pressure.

**Table 1:** Number of participants who acquired single-lineage infections sensitive to VRC01, defined as IC_80_ ≤ 3 µg ml^−1^

	HVTN 703/HPTN 081 (Africa trial)				HVTN 704/HPTN 085 (Americas trial)			
	VRC01 10 mg kg^−1^	VRC01 30 mg kg^−1^	Placebo	Total	VRC01 10 mg kg^−1^	VRC01 30 mg kg^−1^	Placebo	Total
No. HIV-1 diagnosis*	34	24	29	87	37	34	41	112
No. single-lineage infections (Env)**	22	11	17	50	19	26	29	74
No. single VRC01- sensitive lineage	9	4	10	23	6	7	11	24
Subtype	9C	4C	10C		5B, 1F	6B,1F	8B, 1F1, 1F2, 1BF	
No. individuals with variant VRC01 sites	6	3	2	11	1	3	3	7
No. env sequences at timepoint 1 median (range)	33 (3–100)	193 (71–245)	123 (23–369)	71 (3–369)	155 (16–324)	104 (3–379)	181 (27–333)	138 (3–379)
No. env sequences at timepoint 2 median (range)	61 (5–184)	81 (63–216)	80 (4–208)	74 (4–216)	165 (30–192)	112 (28–357)	115 (42–467)	116 (28–467)

*All participants who acquired HIV-1 were included except for 25 participants with sequence data from only a single timepoint.

**Six participants were excluded because their first sequencing timepoint was ≥45 days from the estimated date of acquisition (range 45–73 days)^30^. Of the 47 single-lineage infections with sensitive viruses, 39 occurred by the week 80 visit (21 from HVTN 703; 24 from HVTN 704) and 8 occurred after the week 80 visit (2 from HVTN 703; 6 from HVTN 704).

In total, we analysed 11,730 previously generated *env* sequences from two timepoints (median 108 sequences per participant per timepoint; range 3–379)^3,5,7^. The median time from estimated date of acquisition to the first sequencing timepoint was 18 days (range 7–45 days), with low intraparticipant diversity at this timepoint, consistent with recent acquisition (median 0.05% DNA distance, range 0.02–0.12%). The median interval between sequencing timepoints (trials pooled) was 14 days (range 5–100 days) for placebo and 12 days (range 6–63 days) for the VRC01 treatment group (dose groups and trials pooled). No participants had initiated ART at the time sequencing of plasma virus was conducted.

### Evolution in the VRC01 epitope post acquisition

**Fig. 1:**
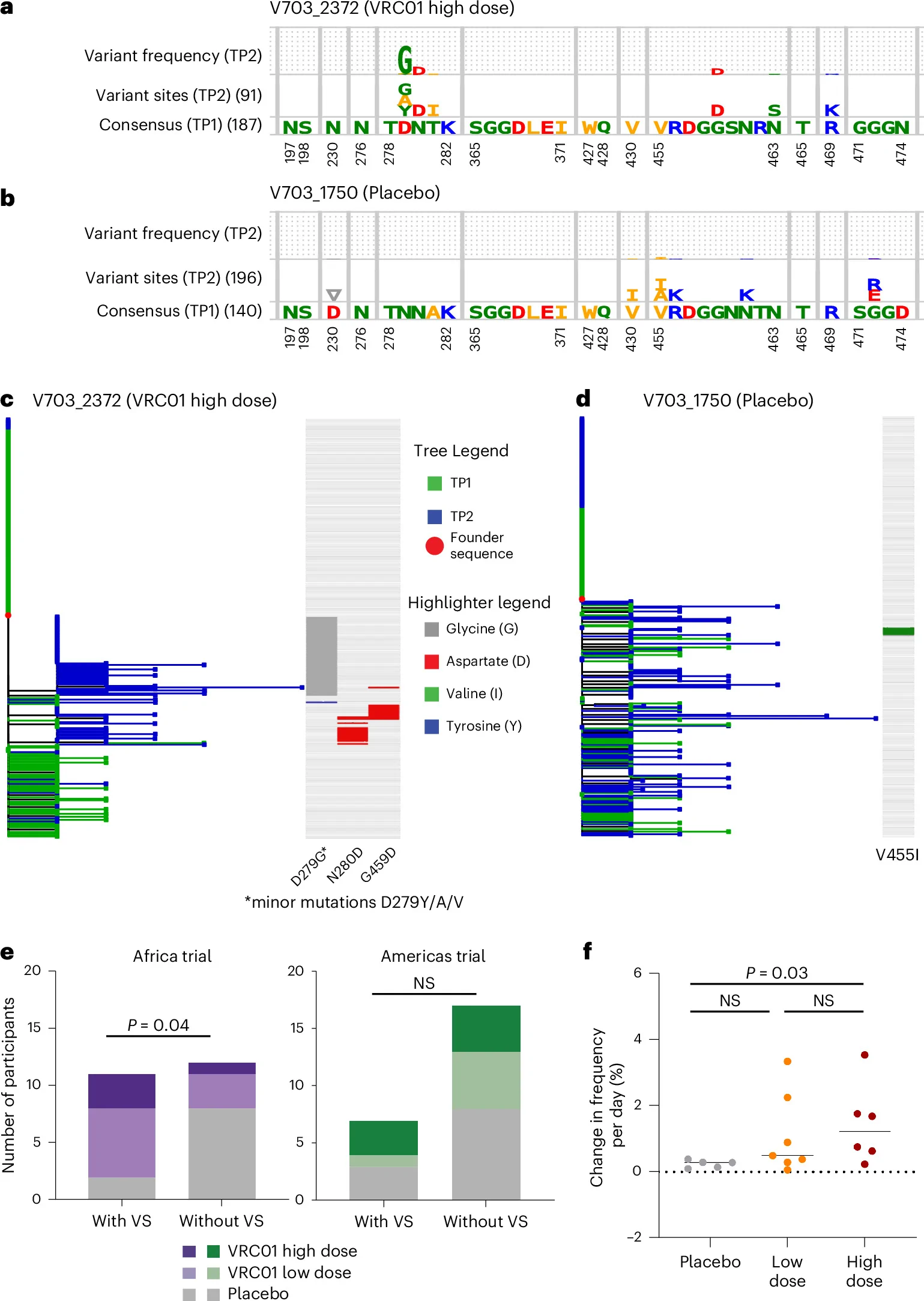
Identification of putative post-acquisition VRC01 escape mutations in individuals with single-lineage infections by VRC01-susceptible viruses (IC_80_ ≤ 3 µg ml^−1^). **a**–**d**, Illustratration of the analytical approaches used to identify amino acid (AA) escape mutations within the 34-residue VRC01 epitope. For illustration, we show a representative from two individuals: VRC01 high-dose recipient (V703_2372, **a** and **c**) and a placebo recipient (V703_1750, **b** and **d**). **a**,**b**, Logoplots comparing AA changes between timepoint 1 (TP1) and timepoint 2 (TP2). Bottom: the TP1 founder lineage consensus sequence. Top: changes in AA frequency in TP2 relative to the consensus, with the height of the letter showing frequency. The middle panel is scaled to enable visualization of low-frequency variant AAs. Sequence counts are shown in parentheses. **c**,**d**, Phylogenetic trees of TP1 (green) and TP2 (blue) sequences. Adjacent highlighter plots display AA changes within the VRC01 epitope relative to the sequence closest to the TP1 consensus, allowing visualization of AA positions that diverged from the TP1 consensus. Sequences identical to the TP1 consensus are shown in light grey, and changes from consensus are shown in colour with the divergent site indicated at the bottom of the plot. V703_2372 had 187 sequences in TP1 and 91 in TP2. V703_1750 had 140 sequences in TP1 and 196 sequences in TP2. **e**, Number of participants with VRC01 variant sites (VS) in the Africa trial (purple) and Americas trial (green). In the Africa trial, there was a significantly higher number of VS in VRC01 recipients (pooled treatment group) than in placebo recipients (*P* = 0.04 two-sided Fisher’s exact test), whereas no significant difference (NS) between treatment and placebo was observed in the Americas trial. **f**, Rate of change in VS prevalence per day between TP1 and TP2, grouped by treatment assignment, with data from each treatment group pooled across trials: placebo (grey), VRC01 low-dose (orange) and VRC01 high-dose (burnt orange). The rate of change in VS prevalence was significantly greater in the VRC01 high-dose group compared with the placebo group (*P* = 0.03, two-sided Mann–Whitney test). Source data

**Extended Data Fig. 1:**
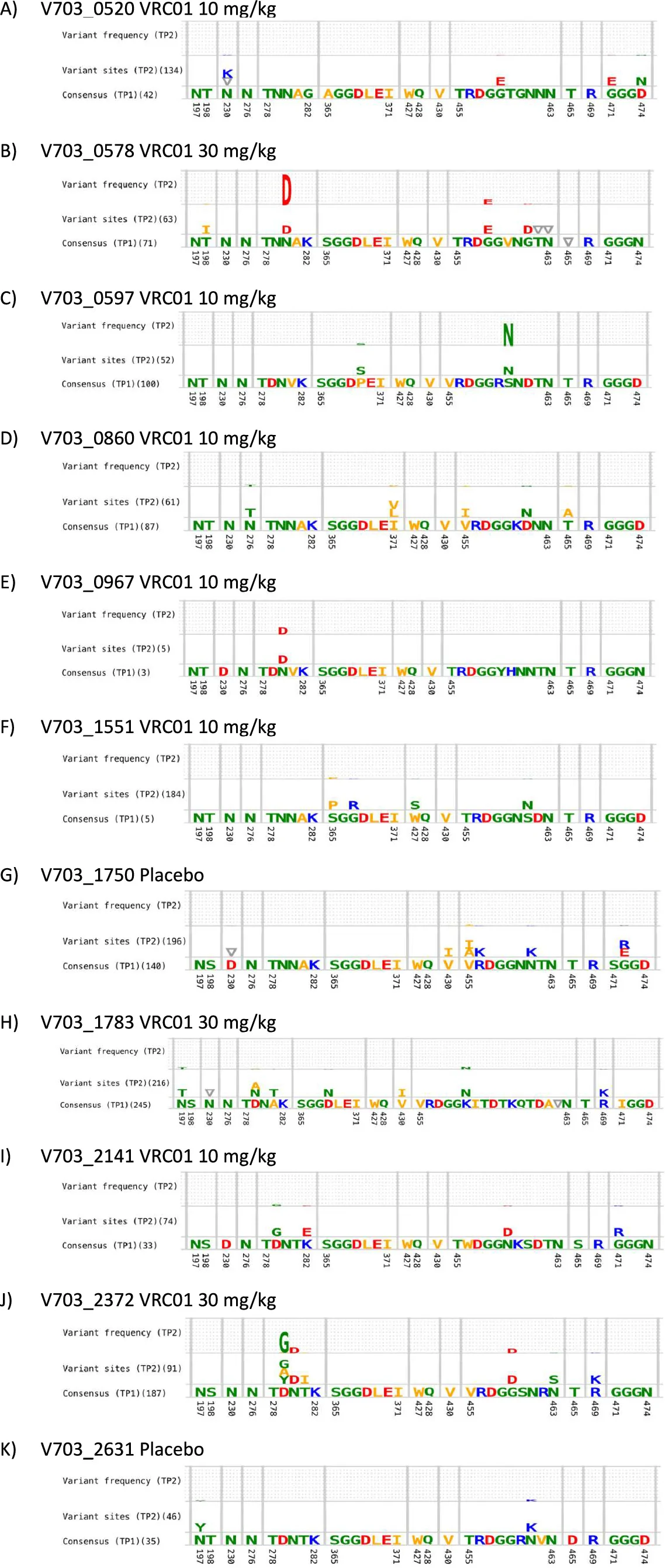
Escape logograms of the 34 VRC01 epitope sites of interest for HVTN 703/HPTN 081 participants. Logograms showing amino acid (AA) changes between time point 1 (TP1) and time point 2 (TP2). The bottom panel presents the founder lineage consensus sequence at TP1, while the top panel shows the frequencies of variants at TP2. The middle panel highlights variant amino acids identified at a site compared to the consensus at TP1 and has been scaled for visibility. Sequence counts at each time point are shown in parentheses.

**Extended Data Fig. 2:**
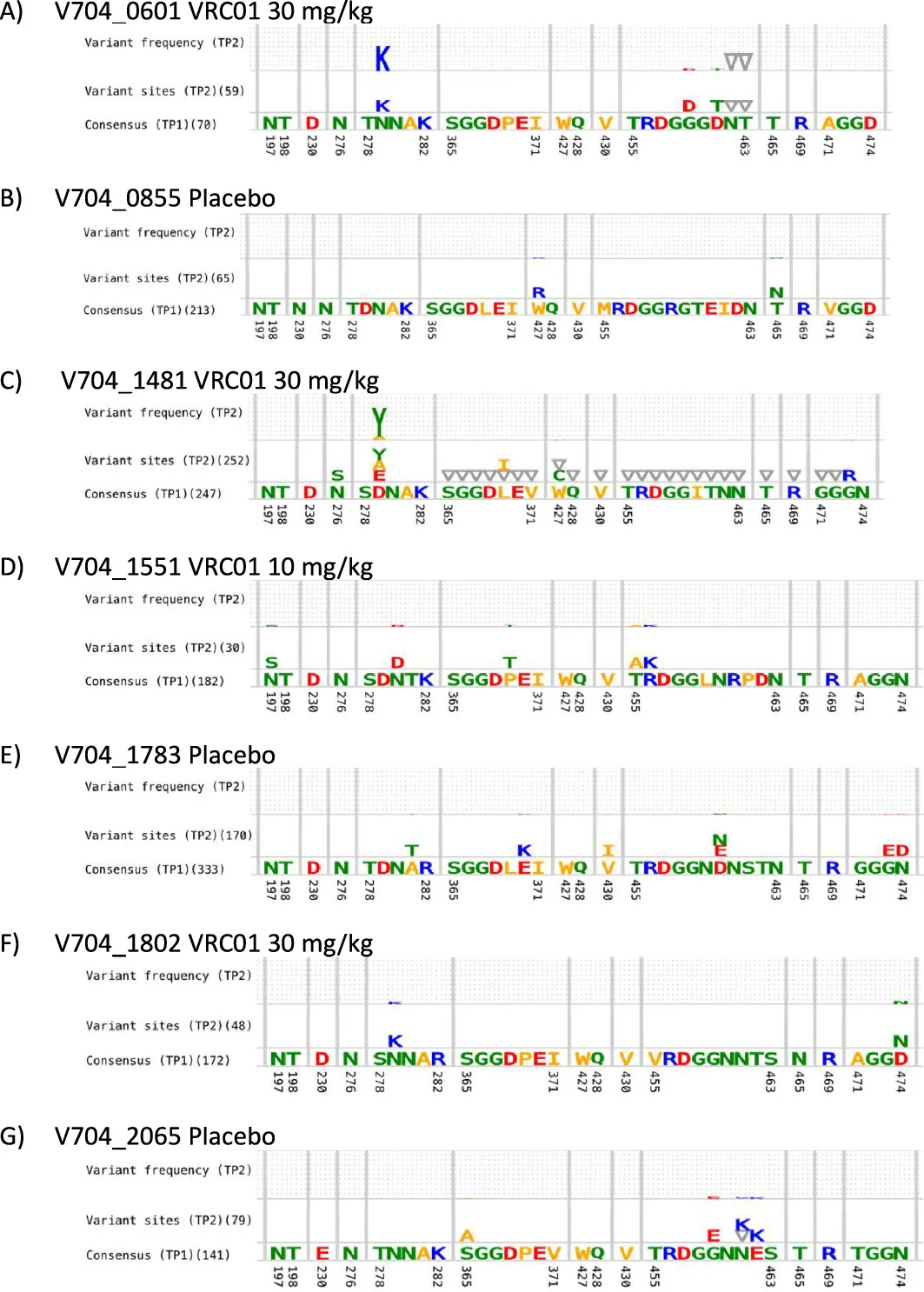
Escape logograms of variant sites in the 34 VRC01 epitope sites of interest for HVTN704/HPTN 085 participants. Logograms showing amino acid (AA) changes between time point 1 (TP1) and time point 2 (TP2). The bottom panel presents the founder lineage consensus sequence at TP1, while the top panel shows the frequencies of variants at TP2. The middle panel highlights variant amino acids identified at a site compared to the consensus at TP1 and has been scaled for visibility. Sequence counts at each time point are shown in parentheses.

**Extended Data Fig. 3:**
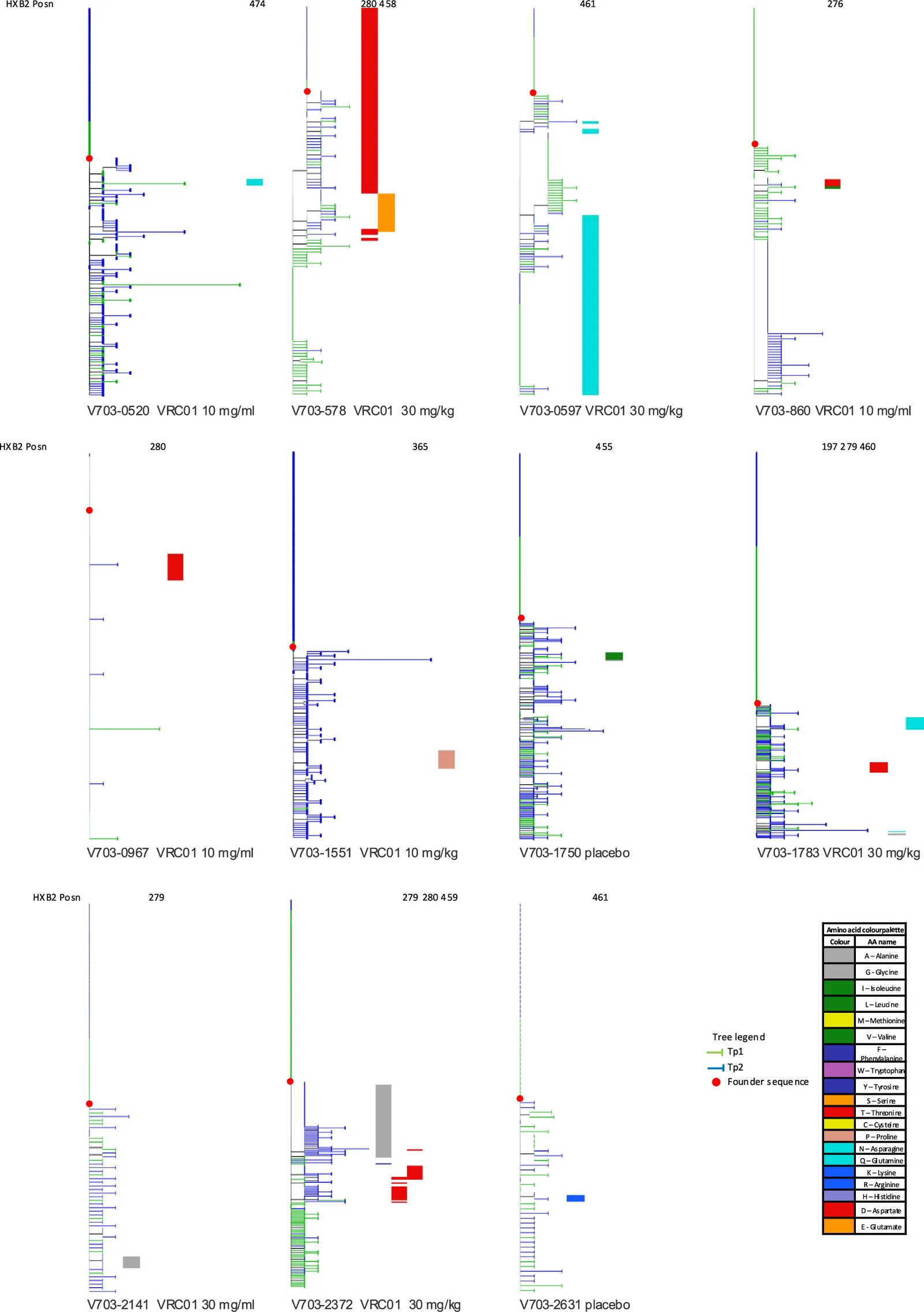
Phylogenetic trees and highlighter plots displaying HVTN 703/HPTN 081 variant sites in the VRC01 epitope (34 residues). Trees show sequences from time point 1 (green) and time point 2 (blue), with branch lengths representing nucleotide changes. Highlighter plots display amino acid changes in the VRC01 epitope sites relative to the sequence closest to a timepoint 1 consensus sequence (red dot), with HXB2 number of variant sites indicated at the top of the plot.

**Extended Data Fig. 4:**
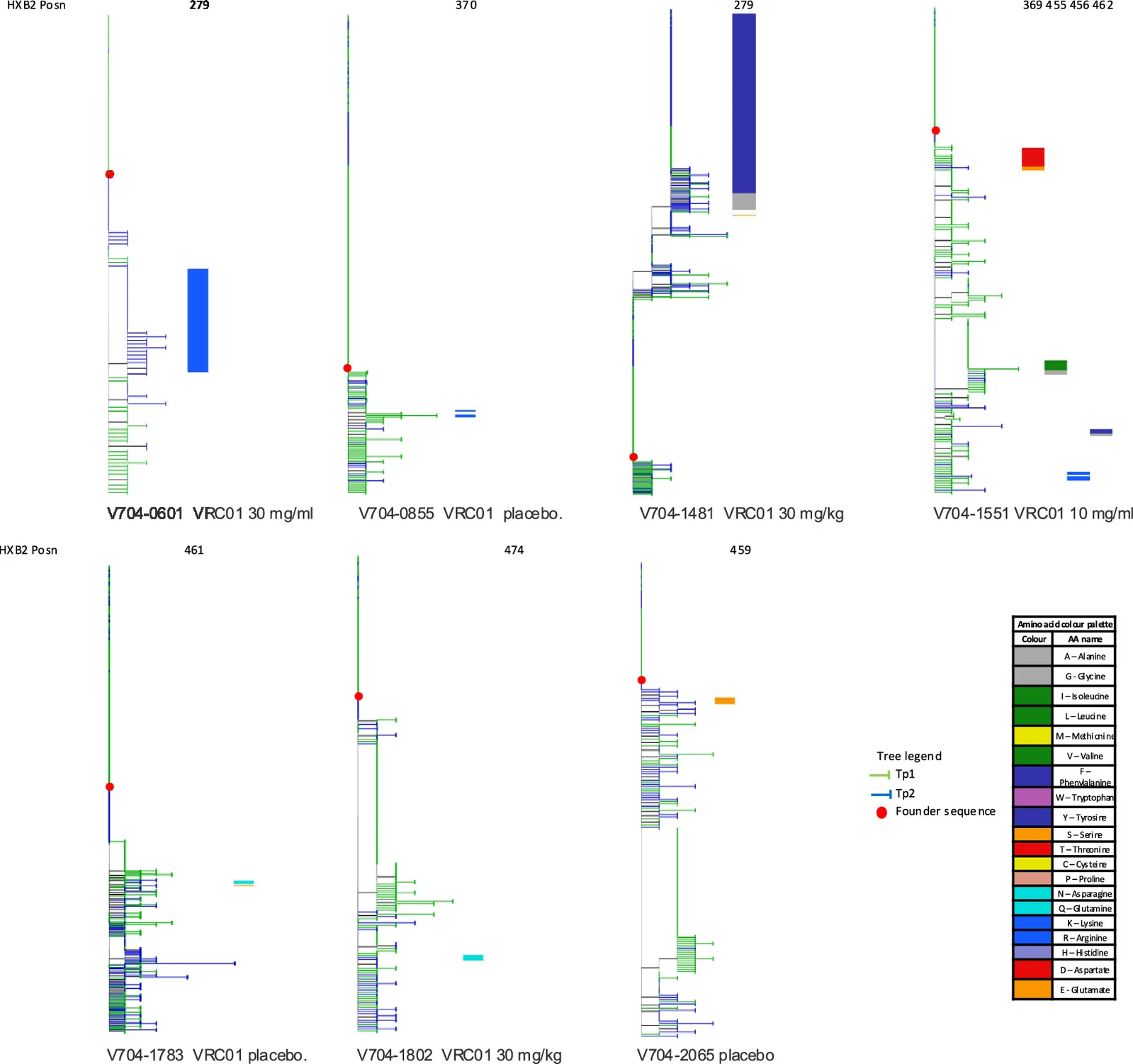
Phylogenetic trees and highlighter plots displaying HVTN 704/HPTN 085 variant sites in the VRC01 epitope (34 residues). Trees show sequences from time point 1 (green) and time point 2 (blue), with branch lengths representing nucleotide changes. Highlighter plots display amino acid changes in the VRC01 epitope sites relative to the sequence closest to a timepoint 1 consensus sequence (red dot), with HXB2 number of variant sites indicated at the top of the plot.

To identify putative VRC01 escape mutations, we tracked changes over time at 34 amino acid sites (variant sites) within the VRC01 epitope (Extended Data Table 1). Two complementary approaches were used. First, escape logograms compared amino acids at timepoint 2 (TP2) to the consensus sequence at timepoint 1 (TP1) (Fig. 1a,b, and Extended Data Figs. 1 and 2). Second, phylogenetic trees with adjacent highlighter plots were used to visualize changes between timepoints and to identify co-occurring amino acid changes (Fig. 1c,d and Extended Data Figs. 3 and 4).

Variant sites were identified in 18 of 47 individuals (Table 1). Among the remaining 29 participants, 24 had at least 129 sequences each, providing 80% confidence in detecting variants present at a frequency of ≥1.25% or higher, and 98% confidence in detecting variants present at a frequency of ≥3%. The remaining 5 participants had fewer sequences (range 27–86), with lower confidence for variant detection at these sequencing depths.

Variant sites were most frequently detected in Env Loop D, with sites 276, 279 and/or 280 identified in 8 individuals, all in the VRC01 groups. In total, 11 variant sites were observed exclusively in the VRC01 group (N197T, D276T/I, D279G/A/Y/K, N280D, S365P, P369S/T, R456K, G458E, K460N, D462Y/G and D474N), 1 variant site (E370K) was observed exclusively in the placebo group, and 3 variant sites (T/V455I/A, G459E/K and D461N/E) were observed in both groups (Extended Data Table 2).

### VRC01 epitope variant frequency increased faster in the high-dose group

In the Africa trial, variant sites were significantly more common among VRC01 recipients (dose groups pooled) than placebo recipients and were detected in 9 of 13 VRC01 recipients compared to 2 of 10 placebo recipients (*P* = 0.04, two-sided Fisher’s exact test) (Fig. 1e). However, there was no difference in frequency of variant sites between placebo and treatment groups (pooled) observed in the Americas trial (Fig. 1e). When the rate of change in variant sites was examined (that is, change in frequency per day, pooled across trials by treatment group), the high-dose group showed a significantly greater rate of change compared with placebo group (*P* = 0.03, two-sided Mann–Whitney test) (Fig. 1f).

### Single and double mutations that confer resistance to VRC01

**Fig. 2:**
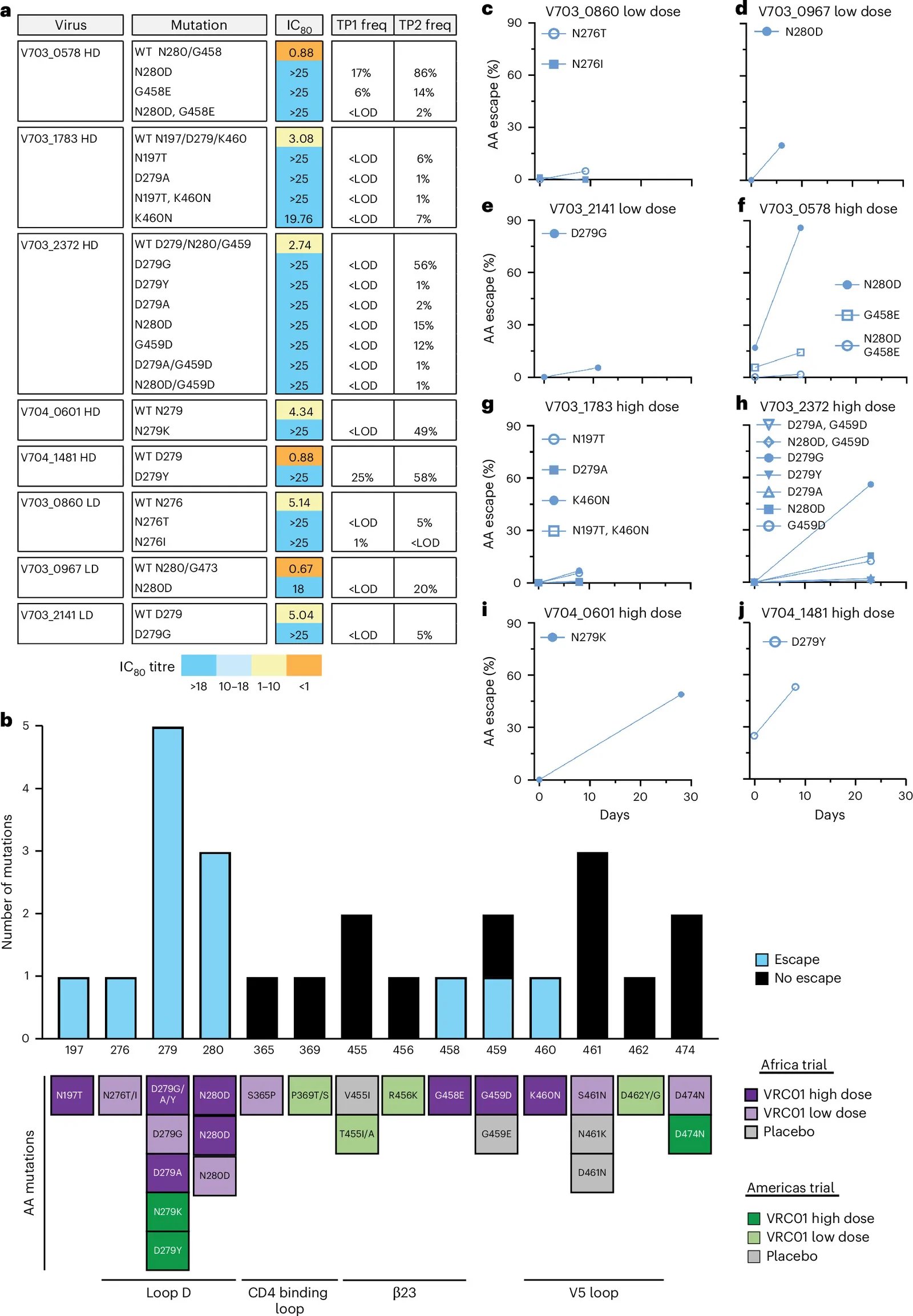
Post-acquisition VRC01 escape mutations. Escape was defined as a greater than 3-fold change in neutralization sensitivity after introduction of the escape mutation into the parental PSV backbone. **a**, Summary table of escape mutations detected in 8 individuals. Each row shows participant ID and trial arm (HD, high-dose; LD, low-dose; placebo), the mutations detected (listed below the wildtype sequence), and the IC_80_ neutralization titres (µg ml^−1^) of the parental PSVs and the corresponding escape mutants colour coded as follows: blue >18 µg ml^−1^, teal 10–18 µg ml^−1^, yellow 1–10 µg ml^−1^, orange <1 µg ml^−1^. The frequency of each mutation at the first (TP1) and second (TP2) timepoint is indicated. <LOD indicates below the limit of detection. **b**, Top: bar chart showing the location of each escape mutation (*x* axis) on gp120, grouped by structural region (Loops D, CD4 binding loop, B23 and V5 loop). Blue bars indicate positions at which escape mutations were detected, and the black bars indicate positions at which variants were observed but did not confer escape. Bottom: specific amino acid substitutions observed are shown as blocks under the bar graph and colour coded by trial group: Africa trial, dark purple (high-dose) and light purple (low dose); Americas Trial, dark green (high-dose) and light green (low-dose). Placebo for both trials is shown in grey. **c**–**j**, Frequency (%) and kinetics of VRC01 escape in each of the 8 participants, plotted relative to days since diagnosis (day 0). Each mutation is represented by a distinct symbol; mutations detected as co-occurring on the same sequence are also shown. Two participants (V703_2141 and V704_1481) received their infusion 25 or 27 days before sequencing (day 0), while the remaining 6 (V703_0578, V703_0860, V703_0967, V703_1783, V703_2372 and V704_0601) received their last VRC01 infusion on the same day as sequencing (day 0). The number of sequences per timepoint is shown in Extended Data Table 2. Variant frequencies were calculated using sequence data processed using the 20% filter^48^, except for participant V703_1783, where the sequence carrying the double mutation (N197T/K460N) was retained using only the 15% filter. Source data

**Extended Data Fig. 5:**
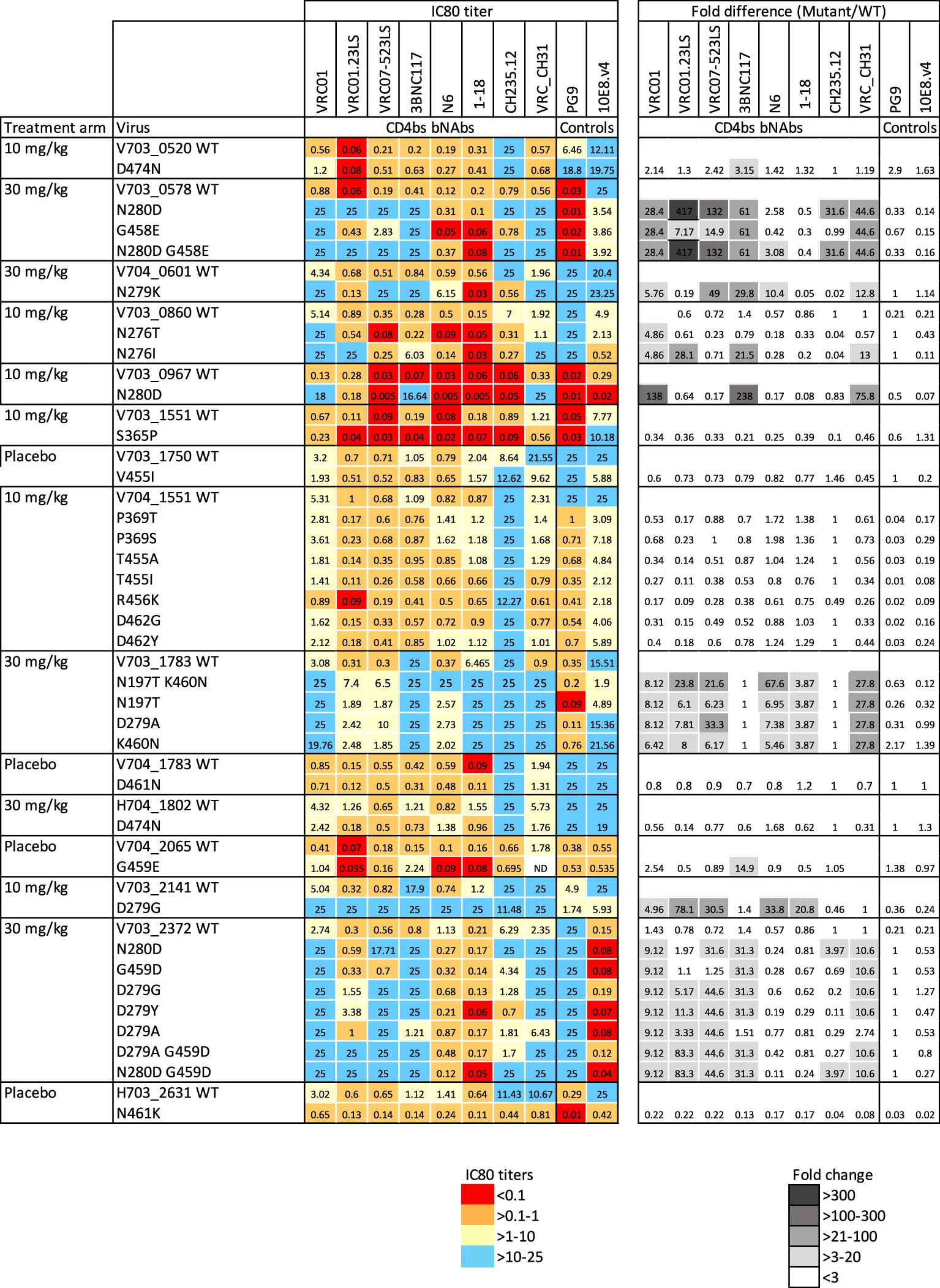
Heat plot of IC80 neutralization titers of pseudoviruses and corresponding mutants. Plot on the lefthand side illustrating the IC80 titers, and the plot on the right-hand side showing the fold difference between the sensitive PSV and the mutant. The eight CD4bs bnAb shown on the top, together with two control bnAbs targeting V2 apex and MPER (PG9 and 10E8v4). VRC01 neutralization sensitivity to V703_0597 and V704_1481 and their corresponding mutants is published3. Cross neutralization was not generated for V703_0597, V704_1481 and V704_0855.

Variant site mutations were introduced into the corresponding VRC01-sensitive PSV clone representing the TF lineage identified at timepoint 1. The impact of the mutations on VRC01 sensitivity was measured in the TZM-bl neutralization assay, which measures antibody-mediated neutralization of HIV-1 after a single round of infection with Env-pseudotyped viruses. Amino acid changes were defined as escape mutations if there was a greater than 3-fold change in IC_80_ (Extended Data Table 2 and Extended Data Fig. 5). Of the 30 single mutants and 4 double mutants, 1 was non-functional (E370K in V704_0855 in the placebo group). The remaining 34 were assayed for VRC01 neutralization sensitivity (Fig. 2 and Extended Data Table 2).

Escape mutations were significantly more likely to emerge post acquisition in the treatment group: Escape was detected in 31% (8/26; 95% CI 15–52%) of individuals who received VRC01, versus none in the placebo group (0/21) (*P* = 0.006, two-sided Fisher’s exact test) (Fig. 2a, Extended Data Table 2 and Extended Data Fig. 5). All participants with VRC01 escape were diagnosed with HIV by the week 80 visit (within 8 weeks of the last VRC01 infusion), with no escape identified in participants who were diagnosed after the week 80 visit. Escape was more common in the Africa trial (46%; 6/13, all subtype C), compared with the Americas trial (15%; 2/13, all subtype B). Among the 8 individuals with escape mutations, 3 were from the low-dose group and 5 from the high-dose group (Fig. 2a,b).

The Loop D region was a hot spot for viral escape mutations, consistent with previous reports^32^, with escape-associated mutations at residues 276, 279 and/or 280 identified in all 8 participants. Mutations at position 279 and 280 were the most frequently observed, arising in 5 and 3 participants, respectively (Fig. 2b). One participant (V703_2372) had mutations at both residues 279 and 280. Toggling of alternative amino acids, probably driven by selection^33^, was observed in two Loop D residues: N276T/I and D279G/Y/A in participants V703_0860 and V703_2372, respectively. Additional escape mutations unique to specific individuals include those in the N197 glycan motif and in the β23-V5 region (G458E, G459D and K460N) (Fig. 2b). We assessed whether sites distal to the epitope co-evolved with escape. In one individual (V703_0578) we identified an A318T substitution, which co-evolved with the G458E escape mutation. Evolution in this site was not observed in any of the 21 placebo participants.

Escape mutations were detected in 3 participants at the first timepoint and generally increased in frequency by the second timepoint, reaching a median frequency of 6% (IQR 1–19%) at timepoint 2 (Fig. 2a,c–j). The variants with mutational toggling (N276T/I and D279G/Y/A) and those carrying double mutations (N280D/G458E, N280D/G458D, N197T/K460N, D279A/G459D), were all present at low frequencies of ≤2%. In 6 of the 8 participants, the final VRC01 infusion coincided with the day of HIV-1 diagnosis (Extended Data Table 2). The remaining 2 participants, 1 in the low-dose group (V703_2141) and 1 in the high-dose group (V704_1481), received their last infusion 25 and 27 days before diagnosis, respectively (Fig. 2c–j and Extended Data Table 2).

### Escape mutations are rare in global reference panels

**Fig. 3:**
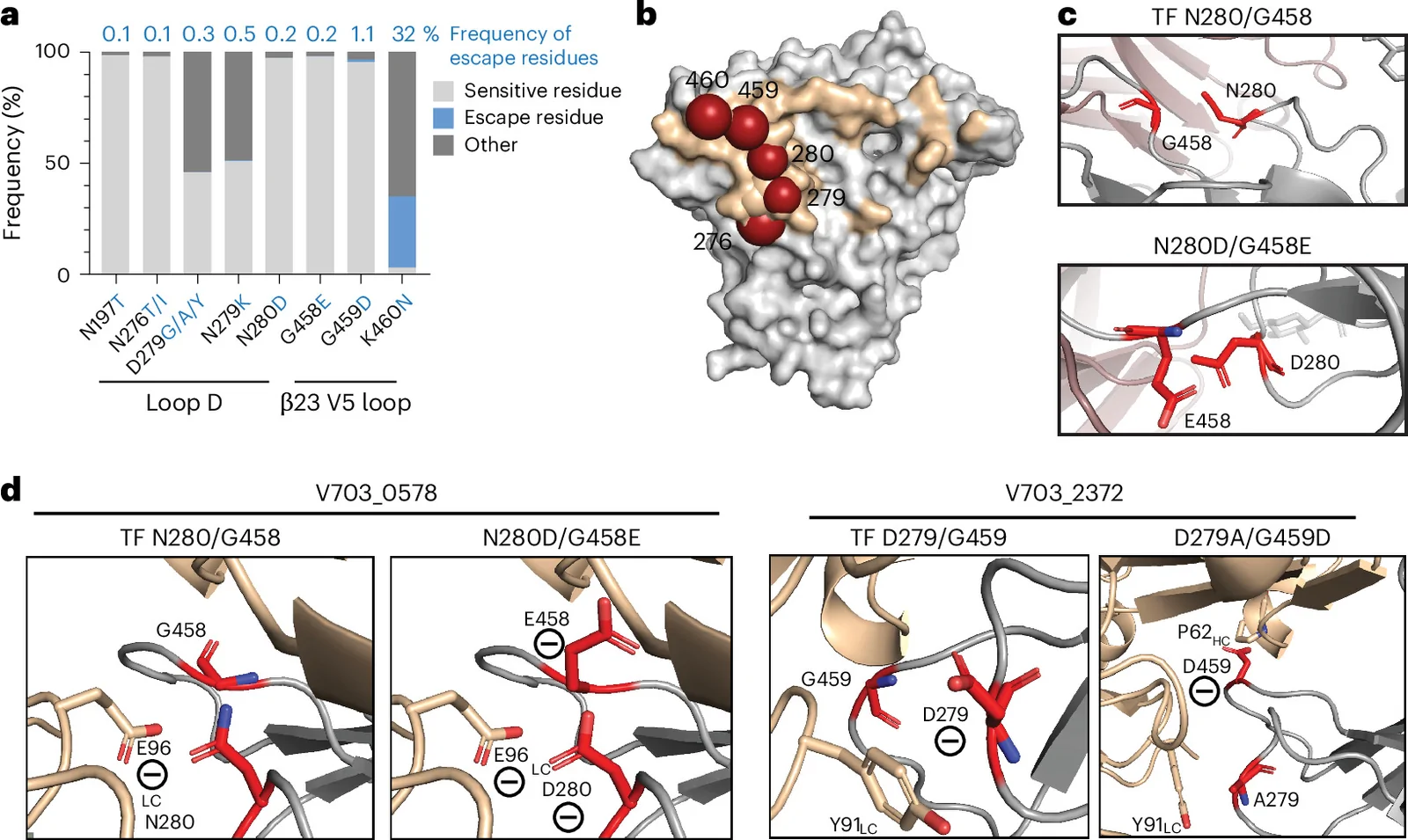
Global frequency of VRC01 escape amino acids and structural mapping on HIV gp120. **a**, Stacked bar charts showing the global frequency of each escape amino acid identified in an alignment of 8,452 filtered HIV sequences from the Los Alamos National Laboratory HIV Sequence Database (https://www.hiv.lanl.gov/). For each escape mutation (*x* axis, grouped by structural region), bars show the proportion of sequences carrying the wildtype residues (AA in the founder lineage, light grey), escape residues (blue) and other (neither wildtype nor escape, dark grey). The global frequency of the escape amino acid is shown above the bars. A low proportion of blue indicates that the escape substitution is rare in globally circulating viruses. **b**, Crystal structure of the gp120 (PDB ID 4LST, grey), with the VRC01 antibody footprint highlighted in wheat colour. Escape mutation positions identified in this study are shown as red spheres, with the residue numbers labelled. **c**,**d**, Close-up images of the interaction between VRC01 (wheat colour) and the HIV gp120 protein (grey). **c**, Top: N280/G458 TFL residues. Bottom: D280 and E458 escape residues. **d**, Charge residues shown as negative symbols indicate predicted introduction of electrostatic repulsion of VRC01, providing a structural explanation for loss of neutralization sensitivity. Participant V703_0578 (left pair) illustrates the TFL (N280/G458) and escape (D280 and E458) residues, with relevant VRC01 contact residue (E96) labelled with negative charge symbol illustrating the electrostatic clashes introduced by the escape mutations. For participant V703_2372 (right pair), the transmitted found residues (D279/G459) and escape residues (A279/D459) are shown, with the VRC01 contact residues P62 and Y91 labelled. Escape mutations were modelled using the mutagenesis tool in PyMOL Molecular Graphics System v.2.4.2 (Schrödinger). Source data

**Extended Data Fig. 6:**
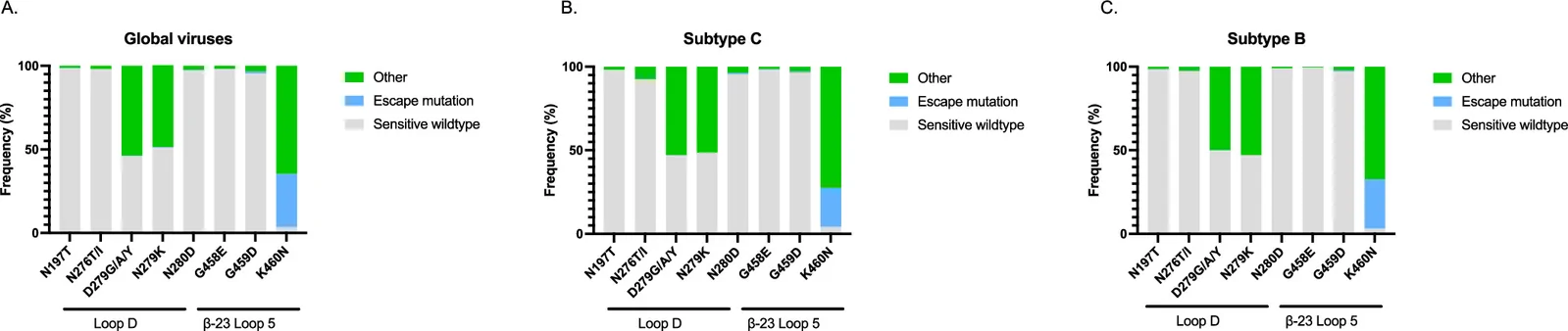
Comparison of the frequency of VRC01 escape amino acids in global, subtype C and subtype B sequences. Stacked bar charts showing global frequency of each escape amino acid in 8,452 filtered HIV sequence alignment from Los Alamos National Laboratory HIV Sequence Database (https://www.hiv.lanl.gov/) (**A**);only subtype C sequences in the alignment (n = 1779), and only subtype **B** sequences (n = 3043) (**C**). For each escape mutations (x-axis, grouped by structural region) bars show the proportion of sequences carrying the wildtype residues (AA in the founder lineage, light grey), escape residues (blue), other (neither wildtype nor escape, dark grey).

Escape was identified at 7 different VRC01 epitope positions. Except for K460N (V703_1783), all mutations were found at a frequency of less than 1% of the HIV sequences in the database (8,452 sequences, www.LANL.gov) (Fig. 3a). The K460N change was a reversion from one relatively uncommon amino acid (11%) to a more common amino acid (32%). The amino acid frequencies of escape mutations in subtype-matched reference panels were similar to the global panel (Extended Data Fig. 6).

### Mechanisms of escape of single and double mutations

We identified three co-occurring mutations, in Loop D and the V5 loop, close to each other on the envelope (N280D/G458E, D279A/G459D and N280D/G459D) (Fig. 3b,c). These mutations were modelled onto a crystal structure of VRC01 bound to a subtype C gp120 protein (PDB ID 4LST) using Pymol v.2.5.2, matching the participant subtype (Fig. 3b). N280D introduces a negative charge that repels the negatively charged E96 residue on the VRC01 light chain, while G458E and G459D introduce negatively charged amino acids with longer side chains. These combined mutations probably disrupt the epitope–paratope interaction (Fig. 3d). In contrast, the D279A/G mutation probably enables escape through shortened side chains and removal of the negative charge (Fig. 3d). Modelling thus suggests that coordinated mutations reshape the viral surface to evade neutralization through alterations in charge and local conformation.

### Some CD4bs bnAbs are unaffected by VRC01 escape

**Fig. 4:**
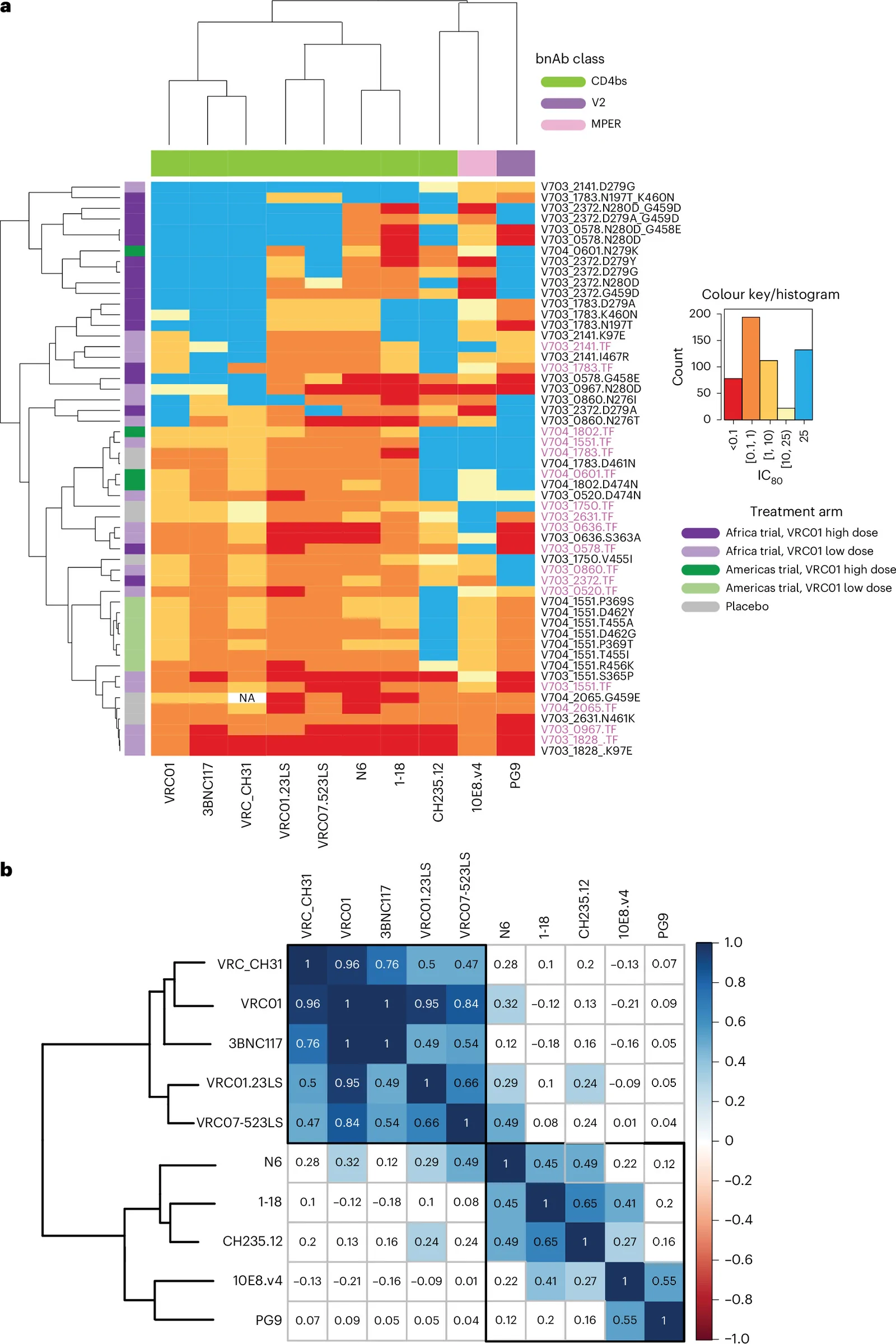
Relationship between escape mutations and bnAb sensitivity. **a**, Heat map of IC_80_ neutralization titres of 54 PSVs (rows) against a panel of 10 bnAbs (columns), yielding 540 PSV bnAb titres in total. The 10 bnAbs comprised 8 CD4bs antibodies (VRC01, 3BNC117, VRC_CH31, VRC01.23LS, VRC07-523LS, N6, 1-18 and CH235.12; labelled as a green bar at the top) and 2 control bnAb targeting the V2 apex epitope (PG9, lavender bar) and the MPER (10E8.v4, pink bar). IC_80_ titres are colour coded (top right). Rows and columns are ordered using hierarchical clustering. The top dendrogram clusters bnAbs by similarity of neutralization profile. The left side dendrogram clusters PSVs on the basis of their neutralization resistance levels, with the most sensitive viruses clustering at the bottom of the heat map (dark red and orange hues) and the more resistant ones at the top (light yellow and blue). Each PSV is listed on the right-hand side, with TFL PSVs in pink and mutant PSVs in black. The coloured bands on the right indicate trial study group: Africa trial high- and low-dose groups in dark and light purple, respectively, and the Americas trial high- and low-dose groups in dark and light green, respectively. Placebo are shown in grey. NA, missing data. **b**, Heat map showing all pairwise correlation coefficients across all pairs of bnAb neutralization titres (columns in the heat map shown in **a**; 10 individual bnAbs for 100 pairs total). Correlation coefficients were calculated using random-effect GLMs to account for the Env backbone as a random effect. White cells indicate no statistical significance, whereas statistically significant correlation coefficients (2-sided ANOVA *P* < 0.05) are shown by coloured cells, colour coded from red (perfect negative correlation) to dark blue (perfect positive correlation). Source data

We screened 17 of the 20 pseudovirus mutants that conferred VRC01 resistance against other VRC01-class CD4bs bnAbs (VRC01.23LS, VRC07-523LS, 3BNC117, N6, VRC_CH31)^27,34,35^, as well as non-VRC01-class CD4bs bnAbs (1-18, CH235.12)^10,36^, generating a neutralization heat map with hierarchical clustering (Fig. 4a and Extended Data Fig. 5). As controls, we included PG9 and 10E8v4, which target the V2 apex and MPER, respectively. To measure pairwise correlations between antibody titres, we performed a random-effect generalized linear model (GLM) regression to account for multiple PSVs (Fig. 4b).

Pairwise GLM coefficients measuring the correlation between each bnAb pair were visualized in a correlation heat map (Fig. 4b). Except for N6, the VRC01-class bnAbs correlated strongly with VRC01 titres (range 0.49–0.96, *P* = 1.2 × 10^−15^ to 3 × 10^−7^, two-sided analysis of variance (ANOVA); Fig. 4b and Extended Data Table 3). In both the hierarchical clustering and correlation heat maps (Fig. 4a,b), 3BNC117 and VRC_CH31 clustered with VRC01. N6, 1-18 and CH235.12 clustered separately, with N6 titres moderately correlated with VRC01 (GLM coefficient of 0.32, *P* = 0.02 by two-sided ANOVA, Fig. 4b and Extended Data Table 3), and 1-18 and CH235.12 showed no significant correlation with VRC01. As previously reported^36^, certain VRC01 escape mutations in Loop D increased neutralization sensitivity to bNAb CH235.12. Substitutions at position 279 increased sensitivity to CH235.12 by 4- to 44-fold in two backbones, V704_601 (N279K) and V703_2372 (D279G/Y/A), respectively. Similarly, the N276T/I mutation in V703_0860 increased sensitivity to CH235.12 by 24-fold.

**Fig. 5:**
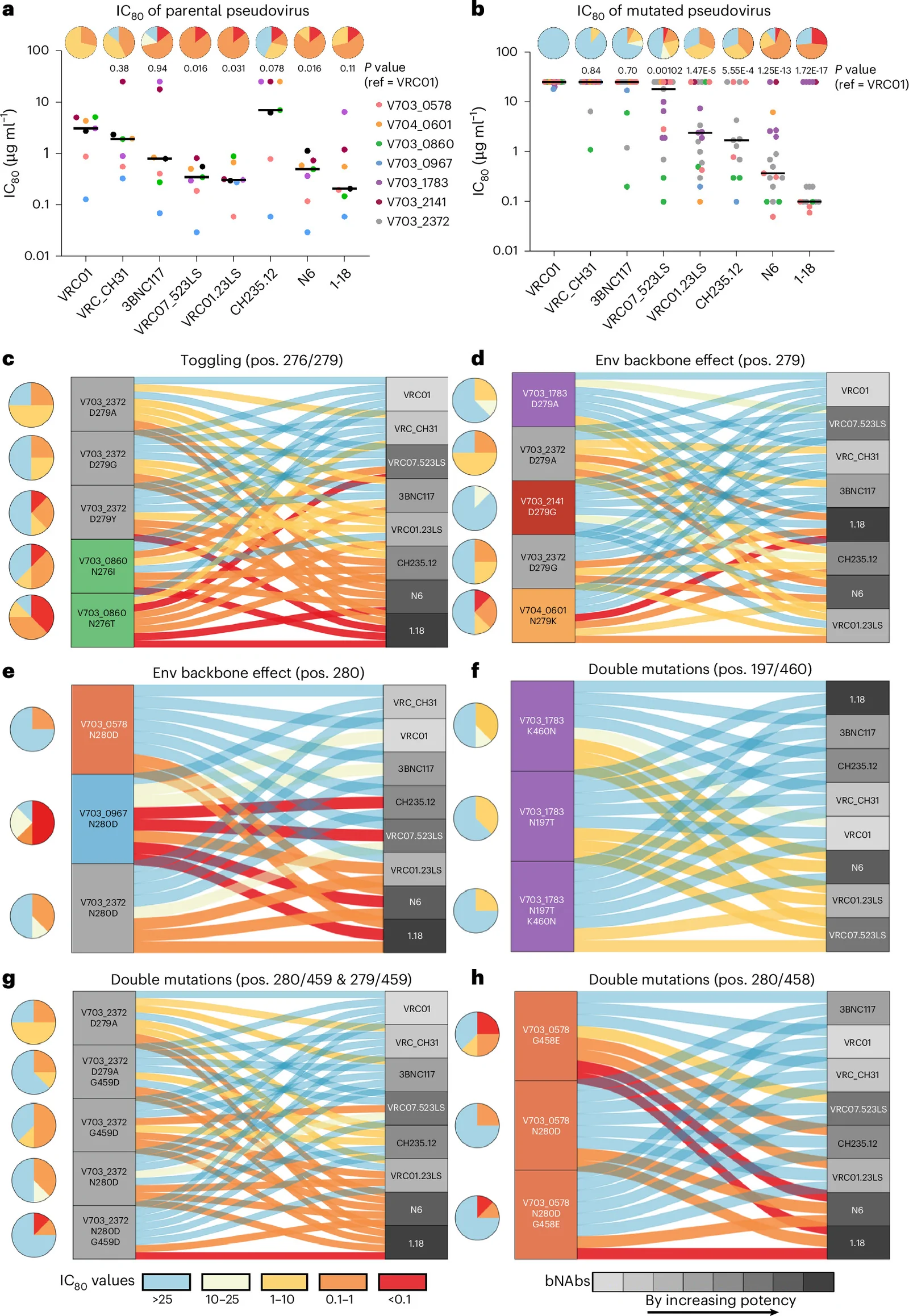
Sensitivity of parental sensitive PSVs and corresponding resistant clones to a bnAb panel, and the impact of Env escape on cross-reactivity. IC_80_ titres (µg ml^−1^) are summarized as pie charts, and with colours consistent across panels ranging from red (highly sensitive) to blue (highly resistant). Similarly, each participant is assigned a unique colour that remains consistent across panels. **a**,**b**, Scatterplots of IC_80_ titres (µg ml^−1^) for 8 bnAbs, shown for 7 parental (TF) PSVs (**a**) and the matched PSVs carrying 19 escape mutations (**b**). Horizontal lines indicate the median IC_80_ (µg ml^−1^), and *P* values are from two-way Wilcoxon test (**a**) and two-sided ANOVA (from GLM regression) comparing each bnAb to the VRC01 reference (**b**). **c**–**h**, Alluvial (Sankey) plots showing the sensitivity of each mutant’s PSV (left) to each bnAb (right), with connecting ribbon colour coded for IC_80_ titres (µg ml^−1^). The IC_80_ titres (µg ml^−1^) for each mutant PSV are summarized as pie charts on the left of each plot. The bnAbs are shaded by their potency to the parental PSVs, with darker shades showing increase in potency, as determined in **a** above; their change in order on the plot reflects the differential impact of the individual mutations. **c**, Effect of amino acid toggling at positions D276A/G/Y and N279I/T on bnAb potency. **d**,**e**, Influence of different viral backbones on bnAb sensitivity. **d**, Mutations in position 279 (D279A in V703_1783 and V703_2372; D279G in V703_2141 and V703_2372; and N279K in V704_0601). **e**, Evaluated mutations in position 280 in backbones V703_0578, V703_0967 and V703_2372. **f**–**h**, Effect of double mutation combinations: V703_1783 with N197T/K460N (**f**); V703_2372 with either D279A/G459D or N280D/G459D (**g**); V703_0578 with N280D/G458E (**h**). All amino acid positions correspond to the HXB2 reference HIV sequence. Source data

We next assessed whether the hierarchical clustering of viruses with VRC01 escape mutations corresponded to the observed reduction in potency of CD4bs-directed antibodies. Figure 5 shows IC_80_ titres of the bnAbs against the parental PSVs (Fig. 5a) and mutated PSVs (Fig. 5b), with the pie charts summarizing their IC_80_ profiles. PSVs that were sensitive to VRC01 were generally also sensitive to other VRC01-class antibodies, with VRC07-523LS, VRC01.23LS and N6 having significantly increased potency compared to VRC01 (false discovery rate (FDR) *q* < 0.01, paired Wilcoxon test after multiple-testing correction).

Figure 5B shows the IC_80_ titres for the same antibodies against PSVs carrying VRC01 escape mutations. Here we excluded PSVs where the parental PSV was resistant to specific antibodies including VRC-CH31 (1 virus), 3BNC117 (2 viruses) or CH235.12 (3 viruses). VRC01 escape mutations had a similar effect on the potency of VRC01, VRC-CH31 and 3BNC117, but did not significantly affect the potency of VRC07-523LS, VRC01.23LS, N6 and 1-18. The bnAb 1-18 showed the greatest resilience to VRC01 resistance mutations, retaining activity with IC_80_ values below 1 µg ml^−1^ against 75% of VRC01 escape mutants.

Overall, the mutations mediating escape from VRC01 also affected most other bnAbs, with the exception of N6 and 1-18 which were largely unaffected by these mutations. We used alluvial plots (Fig. 5c–h) to illustrate the relationship between escape mutations (left side) and bnAbs (right side). Each plot shows pie charts summarizing the IC_80_ profile of a given mutant virus and coloured flows (alluvia) connecting each mutant to the bnAb, coloured by IC_80_ titre. The bnAbs are shaded from light to dark grey on the basis of their overall potency against the escape variants identified in Fig. 5b.

Loop D mutations were most frequently observed (Fig. 2b), but their impact on resistance varied depending on both the specific mutation and the Env backbone in which they occurred (Fig. 5c–e). Furthermore, at positions 276 and 279, alternative amino acids (that is, toggling escape mutations) within the same backbone had different effects on resistance. For example, as shown in Fig. 5c, in the V703_0860 backbone, N276I conferred greater resistance than did N276T, and similarly, in the V703_2372 backbone, D279G or Y conferred greater resistance than did D279A. The impacts of D279A, D279G and N280D were also context dependent (Fig. 5d,e): the N280D mutation conferred resistance to 6/8 bnAbs in the V703_0578 backbone, compared to 4/8 bnAbs in the V703_2372 backbone and only 1/8 bnAbs in the V703_0967 backbone (Fig. 5e). Similar backbone effects were observed for the D279A and D279G mutations (Fig. 5d).

When double mutations were observed, the relative contribution of each mutation varied. In V703_1783, the N197T/K460N double mutant conferred greater resistance to CD4bs antibodies than either single mutation alone (IC_80_ 2.02 and 2.57 µg ml^−1^ for single mutants versus >25 µg ml^−1^ for the double mutant; >10- to >12-fold increase) (Fig. 5f). Similarly, in V703_2372, the double mutants (N280D/G459D) and (D279A/G459D) showed greater resistance than the corresponding single mutants (IC_80_ 0.33 and 0.59 µg ml^−1^ versus >25 µg ml^−1^; >42- to >76-fold increase) (Fig. 5h). In contrast, the V703_0578 N280D/G458E double mutant showed no greater resistance than the N280D single mutant (both being resistant to 6/8 bnAbs) (Fig. 5g).

Regardless of mutation or backbone, bnAbs 1-18 and N6 were generally resilient to VRC01 escape mutations, as indicated by their position towards the bottom of the alluvial plots in 5 out of 6 cases.

## Discussion

The AMP trials provided a unique opportunity to elucidate viral escape pathways under defined antibody pressure immediately post acquisition, a dynamic that is difficult to measure in therapeutic trials where archived resistance can complicate interpretation^26,28,37^. Despite low HIV diversity at transmission which may increase the barrier to escape^19,22^, VRC01 escape mutations emerged in a third of treated individuals. Most clustered in the Loop D region of Env, an important contact site for VRC01-class antibodies^9,10,12,24,34,38^. These mutations reduced the effectiveness of some CD4bs bnAbs, but had a limited effect on more potent antibodies such as N6 and 1-18. The rarity of these mutations in circulating viruses suggests that they impose a fitness cost^15,16,39^. Because VRC01 exposure is transient, resistant variants may not persist long enough to spread; however, this remains an open question. Mapping escape following acute infection provides information relevant to the design of bnAb-based prevention strategies and germline-targeting vaccines, making long-term surveillance of these variants in treatment and prevention studies a priority.

We have previously used long-read deep sequencing data from the AMP trial to elucidate early viral population dynamics following acquisition^21^ and VRC01 pressure on the transmission bottleneck^7^. Here we used these data to investigate escape post acquisition, identifying low-frequency escape variants and co-occurring mutations across gp120. In timepoint 2, 40% of individuals had escape mutations present at a frequency of 2% or lower. Standard approaches with a sequencing depth of ±20 sequences^20^ would have identified these mutations in only 40% of cases (by binomial test), leaving a substantial gap that could obscure emerging resistance in prevention and cure trials. These findings highlight the importance of incorporating deep sequencing protocols into resistance screening, where early identification of low-frequency escape variants is essential for optimizing interventions.

Multiple participants with escape mutations at position 279 and 280 preferred evolutionary escape pathways in acute infection. Similar mutations were identified in viral rebound in people living with HIV (PLWH) treated with ART during chronic infection who subsequently received VRC01 followed by analytical treatment interruption (ATI)^24^, although in these individuals, a larger repertoire of mutations were identified. Some viruses acquired double escape mutations that enhanced resistance, conferring 10- to 76-fold greater cross-resistance to CD4bs bnAbs than either mutation tested alone (Extended Data Fig. 5).

In one individual, the mutation A318T co-evolved with the escape mutation G458E. The G to E change introduces a charged, bulky residue into a constrained binding interface, which probably affects engagement with the CD4bs, and mutations in this position have been associated with a fitness cost^16^. The change from alanine to threonine at position 318 introduces a change from a hydrophobic residue to a polar, uncharged residue which may contribute to compensating for the fitness impact of G458E. Although the precise mechanisms remain unclear, changes in residue 318 have been reported to have some impact on sensitivity to VRC01 (ref. ^8^).

Most CD4bs antibodies in this study belong to the VRC01 class, although each has unique features affecting potency and breadth^9,10,12,34,36,40^. VRC01 escape mutations conferred cross-resistance to most other VRC01-class antibodies, but N6 was less affected probably due to its different binding angle^34^. The highly potent non-VRC01-class CD4bs antibody 1-18 (ref. ^10^) was also largely resilient to VRC01 escape mutations, although mutations at position 197, 279 and 460 increased resistance by 4- to 20-fold in specific backbones (Extended Data Fig. 5). Although these mutations have not previously been reported to affect 1-18 neutralization, they are located in antibody contact sites, and sites 197 and 279 were shown to be under selective pressure in mutational antigenic profiling assays^10^. Overall, bnAb 1-18 retained its potency with a median IC_80_ < 0.1 µg ml^−1^ against VRC01 escaped viruses.

The emergence of viral resistance has been observed in ART-based PrEP trials and bnAb therapeutic trials^26,28,29^. Our findings show that viral escape can also occur in bnAb-mediated PrEP. We identified escape mutations in 3 participants at the time of diagnosis, most probably because these infections occurred after VRC01 levels declined below protective levels^4,6^. In the remaining 5 cases there was no detectable escape at the time of diagnosis; however, VRC01 infusions were inadvertently administered at the diagnostic visit. It is possible that high VRC01 levels during exponential viral expansion in acute infection may have driven de novo escape. It has been hypothesized that bnAbs can suppress replication to undetectable levels, providing a milieu amenable to viral escape. Consistent with this, bar-coded virus challenge studies in non-human primate models showed that prophylactic simian immunodeficiency virus-specific bnAbs can delay diagnosis, with transient viral escape observed in 1 of 6 animals^41^.

In summary, these findings reveal that bnAb-driven escape in PrEP is common, convergent and detectable at an earlier stage than conventional methods permit. Understanding the HIV mutational landscape provides insights into the boundaries of antibody recognition and specific vulnerabilities that may undermine prevention, treatment and remission (functional cure) trials^1,26,42,43^. Collectively, these data underscore the need to account for natural escape pathways to inform selection of bnAb combinations that will raise the barrier to escape. As newer bnAbs with greater potency, broader coverage and longer half-lives enter clinical evaluation, this work provides a framework for mitigating viral resistance and informs optimization of antibody-based prevention strategies capable of constraining HIV’s adaptability.

## Methods

### Ethics statement

This research complies with all relevant ethics regulations. No human material was used in this study. This study used sequences and clinical data generated for the HVTN 703/HPTN 081 and HVTN 704/HPTN 085 studies^3^. All participants gave written informed consent, and the latest protocol version and sample informed consent form is provided (https://doi.org/10.7910/DVN/GDESJI)^44^. The TZM-bl neutralization assay studies at Duke were approved by the Duke University Health System Institutional Review Board (Duke University, protocol no. Pro00093087). The work at the University of Cape Town was approved by the UCT Human Research Ethics Committee (HREC no. 113/2020), and the work at the National Institute of Communicable Diseases was approved by the University of the Witwatersrand Human Research Ethics Committee (protocol no. M201105). The TZM-bl neutralization assay studies at Duke were approved by the Duke University Health System Institutional Review Board (Duke University, protocol no. Pro00093087).

### Study populations

See AMP study^3,44^. In brief, HVTN 703/HPTN 081 and HVTN 704/HPTN 085 were two parallel phase 2b, multicentre, randomized, double-blind, placebo-controlled, proof-of-concept efficacy trials of the broadly neutralizing HIV monoclonal antibody (bnAb) VRC01 in women with a high likelihood of HIV acquisition (HVTN 703/HPTN 081, conducted in sub-Saharan Africa referred to as the Africa Trial) and cisgender men and transgender persons with a high likelihood of HIV acquisition (HVTN 704/HPTN 085, conducted in North America, South America and Switzerland referred to as the Americas Trial)^3^. Participants were healthy adults without HIV who provided written informed consent after an evaluation of their understanding of the protocol. HVTN 704/HPTN 085 enrolled adults aged 18–50 years. HVTN 703/HPTN 081 enrolled adults aged 18–40 years. Study participants were financially compensated for their participation in the study, with the amount varying by geographic location.

Participants received either 10 mg kg^−1^ weight (low dose, LD), 30 mg kg^−1^ (high dose, HD) or saline placebo every 8 weeks and were tested for HIV infection monthly. The earliest RNA-positive plasma sample was sequenced and the dominant infecting viruses (>10% of the population) were evaluated for VRC01 neutralization sensitivity using the TZM-bl assay^3^. Viral sequences were also obtained from a second sampling visit 1–3 weeks later^30,45–47^. In this study, the date of acquisition was assumed to be 7 days before the estimated date of diagnosable acquisition (EDDA) to account for the infection eclipse phase. EDDAs were provided by ref. ^30^ and estimated using a Bayesian posterior distribution, which combined independent inputs from diagnostic timing estimators with combined gag-Δpol and *rev-env-Δnef* sequence-based estimators utilizing Poisson Fitter (2.0)^30,45,46^.

### Sequencing

We used sequences from the earliest RNA-positive plasma sample, as well as a follow-up sequence generated 1–3 weeks later. These sequences were previously generated and are reported in an AMP sieve analysis study^5^, a breakthrough infection study^7^ and an acute HIV transmission study^21^. In summary, sequences were generated using the Pacific Biosciences single-molecule real-time SMRT platform from amplicons derived from cDNA tagged with unique molecular identifiers (UMIs)^48^. Each viral genome was tagged with a UMI during complementary DNA (cDNA) synthesis. Unused cDNA primers were removed and the *rev-env-Δnef* (REN) genomic region (~3 kb; HXB2 position 5,970–9,012) was amplified. To minimize recombination during PCR, a limited number of templates were amplified per PCR reaction. Amplicons were sequenced on the PacBio platform using Sequel instruments. Circular consensus sequences (CCS) were processed into template-specific consensus sequences using custom bioinformatics pipelines, for UMI-containing samples (PORPIDpipeline: https://doi.org/10.5281/zenodo.21430675)^49^. Briefly, CCS reads were filtered for length and quality, then demultiplexed to generate consensus sequences linked to a unique index or sample ID and UMI. Unless otherwise noted, all results reported in this study used the 20% filter to remove artefacts created by carryover by a small amount of UMI-containing cDNA primers.

### Viral characterization for participant selection

For the purposes of this study, we only included participants infected by a single lineage that was sensitive to VRC01 in the AMP trial (IC_80_ ≤ 3 µg ml^−1^)^3,5^. Lineage assignment is described in detail in ref. ^21^. Briefly, two major approaches were taken: the Poisson fitter method applied to the first sequencing timepoint only^45^, and a phylogenetic/distance method which analysed data from all timepoints. The distance/phylogenetic approach generated maximum-likelihood phylogenetic trees from all timepoints pooled. Nucleotide changes from the most common sequence at the first timepoint were visualized using highlighter and matcher plots in Phylobook (https://github.com/MullinsLab/phylobook)^50^. Trees with single clades with low pairwise distances were classified as single-lineage infections, while multiple-lineage infections had more than one clade on the phylogenetic trees, with intraclade distances, after exclusion of recombinant and hypermutated sequences, less than the interclade distances.

### Identification of amino acid variant sites in the VRC01 epitope

We defined the VRC01 epitope as amino acids that were VRC01 contact sites as well as having supporting in vitro evidence demonstrating VRC01 escape (Extended Data Table 1). We also included site 230, which was associated with prevention efficacy in virus sequence sieve analysis in the Americas trial^5^. These sites were in the following domains: C2 region (197, 230); CD4 contact (198, 459, 472–474); Loop D (276, 278–281); CD4 binding loop (365–371); β20, β21/CD4 contacts 427–428, 430; β23/CD4 contacts (456–458); V5 loop (460); loop β24 (465, 469, 471). Variant sites were identified in sequence alignments blinded by treatment group.

All sites were numbered according to HXB2 coordinates (https://www.hiv.lanl.gov/components/sequence/HIV/featuredb/search/feature_search.comp). Intraparticipant changes in VRC01 epitope over time were visualized using escape logograms which illustrate change from the consensus sequence in timepoint one, with the height of the letter showing frequency https://github.com/HughMurrell/EscapeLogos (https://doi.org/10.5281/zenodo.21429943)^51^. We also visualized amino acid differences from the VRC01 epitope using a tool called Mupundu (https://github.com/HIVDiversity/mupundu_public)^52^. Mupundu is written in R and uses the Shiny package and the ggtree package. It takes three input files: a fasta file flagged by timepoint, a tree file in Newick format and a file containing the information about the site of interest (site position, amino acids associated with resistance/sensitivity). From these inputs, Mupundu displays a tree alongside a highlighter plot, showing differences in the VRC01 epitope compared to the sensitive pseudovirus sequence in timepoint 1 (putative transmitted founder lineage sequence). Amino acid residue positions were classified as variant sites (VS) if there were 5 or more viral sequences with an amino acid change at a particular site when compared to the transmitted founder lineage consensus sequence at both timepoints.

### Mutagenesis

Variant sites were introduced into the matched participant TFL virus envelope using site-directed mutagenesis (Agilent QuikChange Lightning or QuikChange Lightning Multi Site-Directed Mutagenesis kits) following manufacturer instructions. Mutations were confirmed by Sanger sequencing. The parental plasmids and corresponding mutants are available on request from penelope.moore@wits.ac.za, with an expected response time of ~1 week.

### PSV production and neutralization assays

PSVs were produced from the TFL and mutant plasmids, and VRC01 sensitivity directly compared using the TZM-bl neutralization assay. For the generation of HIV-1 Env-pseudotyped virus, 293T/17 cells were co-transfected with *env* plasmids and an *env*-deleted backbone vector (pSG3delEnv). The PSV representing the TFL and mutant PSVs were then titrated to determine the optimal dilutions to use in the TZM-bl neutralization assay^53^. Briefly, neutralization was measured as a function of reduction in luciferase reporter gene expression (due to the presence of bnAbs) after a single round of HIV-1 Env-pseudotyped virus infection of TZM-bl target cells. Neutralization titres are expressed as the concentration of the VRC01 drug product at which relative luminescence units (RLUs) were reduced by either 50% (IC_50_) or 80% (IC_80_) relative to virus control wells after subtraction of background RLUs in cell control wells.

### Frequency of escape sites

To determine the frequency of amino acid sites conferring escape in global virus panels, Env amino acid alignments of 8,452 group M HIV-1 sequences were downloaded from the Los Alamos HIV sequence database (http://www.hiv.lanl.gov). This alignment contained only one sequence per participant, with very similar sequences and problematic sequences deleted. For frequencies in HIV subtype B and subtype C, a subset of 3,043 and 1,779 sequences, respectively, were extracted.

### Structural modelling

To visualize the location of the escape mutations of interest, we used a crystal structure of the HIV Env gp120 protein in complex with VRC01 (HIV-1 subtype C strain ZM176.66, PDB ID 4LST). The VRC01 antibody footprint was mapped by determining all residues on the gp120 protein that were within 4 Å of either the heavy or light chain of the antibody. The escape mutations were modelled and the interactions they make with the VRC01 mAb were compared to the original residues at the same location. All analyses were conducted in Pymol v.2.5.2.

### Statistical analysis

This study included all participants that met criteria pre-defined in the statistical analysis plan, and no statistical methods were used to predetermine sample sizes. The comparisons of participant numbers with variant sites between trial and treatment arms were calculated using two-sided Fisher’s exact tests performed in Graphpad Prism v.10. The neutralization titre heat map with hierarchical clustering and the pairwise correlation heat map were generated using the R statistical platform (v.4.2.1). Correlation coefficients between bnAb pairs were calculated using random-effects GLMs for each bnAb pair, with IC_80_ titres as fixed effects and env backbone as random effects. Data distribution was assumed to be normal but this was not formally tested. Data points are shown in Fig. 5a,b. Significance of each GLM coefficient was calculated using ANOVA. Statistical comparisons of the IC_80_ titres of the WT viruses between VRC01 and all other bnAbs were conducted using 2-way paired Wilcoxon tests, and FDR multiple-testing correction was applied with a threshold of *q* < 0.1. Both the Fisher exact and Wilcoxon tests are non-parametric tests, hence no assumptions on the data distributions were needed.

### Reporting summary

Further information on research design is available in the Nature Portfolio Reporting Summary linked to this article.

## Supplementary information

Reporting Summary

## Source data

Source Data Figs. 1–5 and Extended Data Table 2 Statistical source data and consolidated metadata.

## Data Availability

Data pertaining to the AMP participants (for example, treatment assignments, visit dates, neutralization assay readouts and so on) are available on Harvard Dataverse (https://doi.org/10.7910/DVN/GDESJI; https://doi.org/10.7910/DVN/3VG3JO)^44,54^. All individual participant data have been deidentified. The GenBank accession numbers for the HIV Env clones used in the TZM-bl target cell neutralization assay are: HVTN 703/HPTN 081 sequences, ON890939–ON891092; HVTN 704/HPTN 085 sequences, ON980814–ON980967. The GenBank accession numbers for the AMP sequences are ON890939–ON891092; ON980814–ON980967; PX890939-PX026359; ON890939–ON891092; PX184492-PX150096; ON980967-ON980814; OR508960-OR508936; OQ912897-OQ912888.1. Source data are provided with this paper.

## Extended data

**Extended Data Table 1:** List of 34 amino acid positions/sites within the VRC01 epitope that were evaluated for changes over time

HXB2 amino acid position/site	Feature	Envelope Domain	Experiment	References/Citations
197	Glycosylation site	Glycosylation site	Neutralization	^14^
198	Glycosylation site	CD4 contact	Neutralization	^14^
230	Glycosylation site	Glycosylation site, Sieve site	Sieve analysis	^5^
276	VRC01 contact site	Loop D	Neutralization	^13^
278	VRC01 contact site	Loop D	Neutralization	^55^
279	VRC01 contact site	Loop D (CD4 contact)	Neutralization	^13^
280	VRC01 contact site	Loop D (CD4 contact)	Neutralization	^55^
281	VRC01 contact site	Loop D (CD4 contact)	Neutralization	^55^
282	VRC01 contact site	Loop D (CD4 contact)	Neutralization	^34^
365	VRC01 contact site	CD4 Binding loop	Neutralization	^56^
366	VRC01 contact site	CD4 Binding loop	Neutralization	^34^
367	VRC01 contact site	CD4 Binding loop	Neutralization	^34^
368	VRC01 contact site	CD4 Binding loop	Neutralization	^34^
369	VRC01 contact site	CD4 Binding loop	Neutralization	^14^
370	VRC01 contact site	CD4 Binding loop	Neutralization	^34^
371	VRC01 contact site	CD4 Binding loop	Neutralization	^34^
427	VRC01 contact site	β20, β21 (CD4 contacts)	Binding	^14^
428	VRC01 contact site	β20, β21 (CD4 contacts)	Binding	^14^
430	VRC01 contact site	β20, β21 (CD4 contacts)	Binding	^14^
455	VRC01 contact site	β23 (CD4 contacts)	Neutralization	^56^
456	VRC01 contact site	β23 (CD4 contacts)	Neutralization	^13^
457	VRC01 contact site	β23 (CD4 contacts)	Neutralization	^34^
458	VRC01 contact site	β23 (CD4 contacts)	Binding	^14^
459	VRC01 contact site	CD4 contact	Neutralization	^56^
460	VRC01 contact site	V5 Loop (CD4 contact)	Neutralization	^55^
461	VRC01 contact site	V5 Loop (CD4 contact)	Neutralization	^13^
462	VRC01 contact site	V5 Loop	Binding	^14^
463	VRC01 contact site	V5 Loop	Neutralization	^13^
465	VRC01 contact site	V5 Loop β24	Binding	^14^
469	VRC01 contact site	V5 Loop β24 (CD4 contact)	Binding	^14^
471	Glycosylation site	β24 (CD4 contact)	Neutralization	^55^
472	VRC01 contact site	CD4 contact	Binding	^14^à
473	VRC01 contact site	CD4 contact	Neutralization	^56^
474	VRC01 contact site	CD4 contact	Neutralization	^34^

**Extended Data Table 2:** Metadata of participants with variant sites

Participant	Treatment Arm	Infusion to TP1 (days)	TP1-TP2 (days)	Sequence count TP1	Sequence count TP2	Variant site	VRC01 Sensitive/Resistant	Variant seq count TP1	Variant seq count TP2	Variant frequency TP1	Variant frequency TP2
V703_0520	10mg/kg	28	23	42	134	D474N	Sensitive	1	2	2.4%	1.5%
V703_0578	30mg/kg	0	9	71	63	N280D	Resistant	12	55	16.9%	87.3%
						G458E	Resistant	4	9	5.6%	14.3%
						N280D,G458E	Resistant	0	1	0	1.6%
V703_0597	10mg/kg	0	10	100	52	S461N	Sensitive	41	33	41.0%	63.5%
V703_0860	10mg/kg	0	8	87	61	N276T	Resistant	0	3	0	4.9%
						N276I	Resistant	1	0	1.1%	0
V703_0967	10mg/kg	0	8	3	5	N280D	Resistant	0	1	0	20.0%
V703_1551	10mg/kg	26	63	5	184	S365P	Sensitive	0	8	0	4.3%
V703_1750	Placebo	28	8	140	196	V455I	Sensitive	0	6	0	3.1%
						V455A	Sensitive	0	1	0	0.5%
V703_1783	30mg/kg	0	8	245	216	N197T	Resistant	0	12	0	5.6%
						D279A	Resistant	0	2	0	0.9%
						N197T,K460N	Resistant	0	1	0	0.5%
						K460N	Resistant	0	15	0	6.9%
V703_2141	10mg/kg	25	11	33	74	D279G	Resistant	0	4	0	5.4%
V703_2372	30mg/kg	0	23	187	91	D279G	Resistant	0	51	0	56.0%
						D279Y	Resistant	0	1	0	1.1%
						D279A	Resistant	0	2	0	2.2%
						N280D	Resistant	0	14	0	15.4%
						G459D	Resistant	0	11	0	12.1%
						D279A/G459D	Resistant	0	1	0	1.1%
						N280D/G459D	Resistant	0	1	0	1.1%
V703_2631	Placebo	0	15	35	46	N461K	Sensitive	0	2	0	4.3%
V704_0601	30mg/kg	0	28	70	59	N279K	Resistant	0	29	0	49.2%
V704_0855	Placebo	28	5	213	65	E370K	*NA	3	0	1.4%	0
V704_1481	30mg/kg	27	8	247	252	D279Y	Resistant	61	146	24.7%	57.9%
V704_1551	10mg/kg	0	6	182	30	P369T	Sensitive	9	1	4.9%	3.3%
						P369S	Sensitive	1	0	0.5%	0
						T455I	Sensitive	6	0	3.3%	0
						T455A	Sensitive	1	1	0.5%	3.3%
						R456K	Sensitive	2	1	1.1%	3.3%
						D462Y	Sensitive	2	0	1.1%	0
						D462G	Sensitive	1	0	0.5%	0
V704_1783	Placebo	0	20	333	170	D461N	Sensitive	1	2	0.3%	1.2%
V704_2065	Placebo	28	28	141	79	G459E	Sensitive	0	3	0	3.8%

*Pseudovirus with E370K were not functional

PtID, Participant study identifier.

Source data

**Extended Data Table 3:** Correlation co-efficients calculated from random effects generalized linear model of Envelope backbones

bNAb 1	bNAb 2	Corr. Coeff. (95% CI)	P-value
VRC01	VRC01.23LS	0.95 (0.74, 1)	9.7E-11
	VRC07-523LS	0.84 (0.56, 1)	2.3E-07
	3BNC117	1 (0.86, 1)	1.9E-14
	N6	0.32 (0.05, 0.59)	0.02
	VRC_CH31	0.96 (0.79, 1)	1.3E-15
VRC01.23LS	VRC07-523LS	0.66 (0.48, 0.85)	1.9E-09
	3BNC117	0.49 (0.26, 0.76)	8.5E-05
	N6	0.29 (0.12, 0.48)	1.8E-03
VRC07-523LS	3BNC117	0.54 (0.3, 0.79)	3.8E-05
	N6	0.49 (0.33, 0.65)	1.0E-07
3BNC117	VRC_CH31	0.76 (0.63, 0.88)	2.1E-17

## Code availability

The scripts used in this study are openly available from GitHub under the standard MIT license at https://github.com/HughMurrell/EscapeLogos (https://doi.org/10.5281/zenodo.21429943)^51^ and https://github.com/HIVDiversity/mupundu_public (https://doi.org/10.5281/zenodo.21432653)^52^.
