# Supplementary material for: Prophylactic treatment with broadly neutralizing antibody VRC01 selects for escape mutants after infection in Antibody-Mediated Prevention trials
Source: Nat Microbiol. 2026 Sep 28;11(10):2694–706. doi: 10.1038/s41564-026-02467-4 (PMC13716832; doi:10.1038/s41564-026-02467-4)
Supplement: Supplementary file 1 [file 41564_2026_2467_MOESM1_ESM.pdf]

nature portfolio

Corresponding author(s): Carolyn Williamson (carolyn.williamson@uct.ac.za) James I. Mullins (jmullins@uw.edu) Penny Moore (pennym@nicd.ac.za)

Last updated by author(s): Jul 10, 2026

Reporting Summary

Nature Portfolio wishes to improve the reproducibility of the work that we publish. This form provides structure for consistency and transparency in reporting. For further information on Nature Portfolio policies, see our Editorial Policies and the Editorial Policy Checklist.

Statistics

For all statistical analyses, confirm that the following items are present in the figure legend, table legend, main text, or Methods section.

n/a	Confirmed	
☐	☒	The exact sample size (*n*) for each experimental group/condition, given as a discrete number and unit of measurement
☐	☒	A statement on whether measurements were taken from distinct samples or whether the same sample was measured repeatedly
☐	☒	The statistical test(s) used AND whether they are one- or two-sided<br>*Only common tests should be described solely by name; describe more complex techniques in the Methods section.*
☐	☒	A description of all covariates tested
☐	☒	A description of any assumptions or corrections, such as tests of normality and adjustment for multiple comparisons
☐	☒	A full description of the statistical parameters including central tendency (e.g. means) or other basic estimates (e.g. regression coefficient) AND variation (e.g. standard deviation) or associated estimates of uncertainty (e.g. confidence intervals)
☐	☒	For null hypothesis testing, the test statistic (e.g. *F*, *t*, *r*) with confidence intervals, effect sizes, degrees of freedom and *P* value noted<br>*Give P values as exact values whenever suitable.*
☒	☐	For Bayesian analysis, information on the choice of priors and Markov chain Monte Carlo settings
☐	☒	For hierarchical and complex designs, identification of the appropriate level for tests and full reporting of outcomes
☐	☒	Estimates of effect sizes (e.g. Cohen's *d*, Pearson's *r*), indicating how they were calculated

Our web collection on statistics for biologists contains articles on many of the points above.

Software and code

Policy information about availability of computer code

Data collection	VRC01 and other CD4 binding site neutralization data of mutants and parental viruses was generated by the NICD laboratory using the PerkinElmer Victor X Luminometer for data collection using the PerkinElmer 2030 software.
Data analysis	The following computations tools were used:<br><br>EscapeLogos: https://github.com/HughMurrell/EscapeLogos.git<br>Mupundu: https://github.com/HIVDiversity/Mupundu_public<br><br>For structural modeling: Pymol v2.5.2.<br><br>For statistical analysis: Graphpad Prism version 10 (Graphpad Software, USA); R statistical platform, version 4.2.1<br><br>Sequence alignment: Geneious Prime 2025.2.2

For manuscripts utilizing custom algorithms or software that are central to the research but not yet described in published literature, software must be made available to editors and reviewers. We strongly encourage code deposition in a community repository (e.g. GitHub). See the Nature Portfolio guidelines for submitting code & software for further information.

Data

Policy information about availability of data

All manuscripts must include a data availability statement. This statement should provide the following information, where applicable:

- Accession codes, unique identifiers, or web links for publicly available datasets
- A description of any restrictions on data availability
- For clinical datasets or third party data, please ensure that the statement adheres to our policy

Data pertaining to the AMP participants (e.g., treatment assignments, visit dates, neutralization assay readouts, etc.) are available on Harvard Dataverse (https://doi.org/10.7910/DVN/GDESJI; https://doi.org/10.7910/DVN/3VG3JO). All individual participant data have been deidentified. The GenBank accession numbers for the HIV Env clones used in the TZM-bl target cell neutralization assay are: HVTN 703/HPTN 081 sequences, ON890939–ON891092; HVTN 704/HPTN 085 sequences, ON980814–ON980967. The GenBank accession number for the AMP sequences are ON890939–ON891092; ON980814–ON980967; PX890939-PX026359; ON890939-ON891092; PX184492-PX150096 ON980967- ON980814; OR508960- OR508936; OQ912897-OQ912888.1.

Research involving human participants, their data, or biological material

Policy information about studies with human participants or human data. See also policy information about sex, gender (identity/presentation), and sexual orientation and race, ethnicity and racism.

Reporting on sex and gender	The original HVTN 703/HPTN 081 and HVTN 704/HPTN 085 trials were two parallel phase 2b, multicenter, randomized, double-blind, placebo-controlled, proof-of-concept efficacy trials of the broadly neutralizing HIV monoclonal antibody (bnAb) VRC01 in women with a high likelihood of HIV acquisition (HVTN 703/HPTN 081, conducted in sub-Saharan Africa referred to as the Africa Trial) and cisgender men and transgender persons with a high likelihood of HIV acquisition (HVTN 704/HPTN 085, conducted in North America, South America, and Switzerland referred to as the Americas Trial).
Reporting on race, ethnicity, or other socially relevant groupings	NA for the current study
Population characteristics	Participants in the original trials were healthy adults without HIV who provided written informed consent after an evaluation of their understanding of the protocol. HVTN 704/HPTN 085 enrolled adults aged 18 to 50 years. HVTN 703/HPTN 081 enrolled adults aged 18 to 40 years
Recruitment	NA for the current study
Ethics oversight	Ethics Statement. This research complies with all relevant ethical regulations. No human material was used in this study. This study used sequences and clinical data generated for the HVTN 703/HPTN 081 and HVTN 704/HPTN 085 studies3. All participants gave written informed consent and the latest protocol version and sample informed consent form is provided (https://doi.org/10.7910/DVN/GDESJI)44. The TZM-bl neutralization assay studies at Duke were approved by the Duke University Health System Institutional Review Board (Duke University, protocol no.Pro00093087). The work at the University of Cape Town was approved by the UCT Human Research Ethics Committee (HREC no. 113/2020), and the work at National Institute of Communicable Diseases was approved by the University of the Witwatersrand Human Research Ethics Committee (protocol no. M201105). The TZM-bl neutralization assay studies at Duke were approved by the Duke University Health System Institutional Review Board (Duke University, protocol no.Pro00093087).

Note that full information on the approval of the study protocol must also be provided in the manuscript.

Field-specific reporting

Please select the one below that is the best fit for your research. If you are not sure, read the appropriate sections before making your selection.

☒ Life sciences ☐ Behavioural & social sciences ☐ Ecological, evolutionary & environmental sciences

For a reference copy of the document with all sections, see nature.com/documents/nr-reporting-summary-flat.pdf

Life sciences study design

All studies must disclose on these points even when the disclosure is negative.

Sample size	We looked at all available data: 199 participants. 47 met criteria for inclusion into the study.
Data exclusions	All participants in AMP who acquired HIV-1 were included except for 25 participants with sequence data from only a single time point. A further 69 were excluded as the virus was resistant to VRC01 (IC80>3 ug/ml); 6 were excluded because their first sequencing time point was ≥45 days from the estimated date of acquisition (range 45-73 days); and 77 were excluded a they were multilineage infections.
Replication	Neutralization data was generated as part of the clinical trial, and is published by Corey et al. NEJM, 2021. Data was generated using the TZM-bl assay that has been formally optimized and validated, including equivalency between the NICD and Duke laboratory. To assay for VRC01

	sensitivitiy, pre-titrated dose of virus was incubated with serial 3-fold dilutions of the VRC01 drug product in duplicate. This method is described in detail by Corey et al, NEJM 2021.<br><br>Neutralization assays were run in duplicate, and all replicates were within three fold. Positive and negative controls were included in all runs and both antibodies and virus stocks were quality controlled by comparison to published data.
Randomization	N/A
Blinding	Variant sites were identified in sequence alignments blinded by treatment group

Reporting for specific materials, systems and methods

We require information from authors about some types of materials, experimental systems and methods used in many studies. Here, indicate whether each material, system or method listed is relevant to your study. If you are not sure if a list item applies to your research, read the appropriate section before selecting a response.

Materials & experimental systems

n/a	Involved in the study
☐	☒ Antibodies
☐	☒ Eukaryotic cell lines
☒	☐ Palaeontology and archaeology
☒	☐ Animals and other organisms
☐	☒ Clinical data
☒	☐ Dual use research of concern
☒	☐ Plants

Methods

n/a	Involved in the study
☒	☐ ChIP-seq
☒	☐ Flow cytometry
☒	☐ MRI-based neuroimaging

Antibodies

Antibodies used	VRC01, 3BNC117, VRC-CH31, VRC01.23LS, VRC07.523LS, N6, 1-18, CH235.12, 10E8.v4, PG9<br><br>Antibody plasmids were synthesized from publicly available sequences and generated in-house. These were tested in the TZM-bl neutralization assay at a starting concentration of 25µg/ml per assay.
Validation	Antibodies were cloned, expressed and quality controlled according to published IC50 data. Antibodies were validated by running neutralization assays against each HIV-1 Env pseudotyped virus and comparing virus/antibody data with previous results publicly available on repositories such as CATNAP.

Eukaryotic cell lines

Policy information about cell lines and Sex and Gender in Research

Cell line source(s)	Cells were provided by Dr David Montefiori, Duke University. HEk 2293/17 cells were obtained from the American Type Culture Collection (ATCC) while the TZM-bl cells were sourced from the NIH AIDS Research and Reference Reagent Program
Authentication	No additional authentication procedures were done except to confirm the morphology and adherence patterns of the cells under the microscope. The cells also performed as expected in virus growth assays. The laboratory used these cells to successfully participate in external proficiency panels
Mycoplasma contamination	Cell line tested negative for mycoplasma
Commonly misidentified lines (See ICLAC register)	None

Clinical data

Policy information about clinical studies

All manuscripts should comply with the ICMJE guidelines for publication of clinical research and a completed CONSORT checklist must be included with all submissions.

Clinical trial registration	ClinicalTrials.gov numbers, NCT02716675 and NCT02568215
Study protocol	PMID: 33730454
Data collection	*Describe the settings and locales of data collection, noting the time periods of recruitment and data collection.*
Outcomes	*Describe how you pre-defined primary and secondary outcome measures and how you assessed these measures.*

Plants

Seed stocks	N/A
Novel plant genotypes	N/A
Authentication	N/A
